# Early glycolytic reprogramming controls microglial inflammatory activation

**DOI:** 10.1186/s12974-021-02187-y

**Published:** 2021-06-09

**Authors:** Junjie Cheng, Rong Zhang, Zhirou Xu, Youliang Ke, Renjuan Sun, Huicui Yang, Xiaohu Zhang, Xuechu Zhen, Long-Tai Zheng

**Affiliations:** Jiangsu Key Laboratory of Neuropsychiatric Diseases and College of Pharmaceutical Sciences, Soochow University, Suzhou, 215123 Jiangsu China

**Keywords:** Glycolytic inhibitors, Microglial cells, Neuroinflammation, NF-κB, 2-DG

## Abstract

**Background:**

Microglial activation-mediated neuroinflammation plays an important role in the progression of neurodegenerative diseases. Inflammatory activation of microglial cells is often accompanied by a metabolic switch from oxidative phosphorylation to aerobic glycolysis. However, the roles and molecular mechanisms of glycolysis in microglial activation and neuroinflammation are not yet fully understood.

**Methods:**

The anti-inflammatory effects and its underlying mechanisms of glycolytic inhibition in vitro were examined in lipopolysaccharide (LPS) activated BV-2 microglial cells or primary microglial cells by enzyme-linked immunosorbent assay (ELISA), quantitative reverse transcriptase-polymerase chain reaction (RT-PCR), Western blot, immunoprecipitation, flow cytometry, and nuclear factor kappa B (NF-κB) luciferase reporter assays. The anti-inflammatory and neuroprotective effects of glycolytic inhibitor, 2-deoxoy-d-glucose (2-DG) in vivo were measured in the 1-methyl-4-phenyl-1,2,3,6-tetrahydropyridine (MPTP)-or LPS-induced Parkinson’s disease (PD) models by immunofluorescence staining, behavior tests, and Western blot analysis.

**Results:**

We found that LPS rapidly increased glycolysis in microglial cells, and glycolysis inhibitors (2-DG and 3-bromopyruvic acid (3-BPA)), siRNA glucose transporter type 1 (Glut-1), and siRNA hexokinase (HK) 2 abolished LPS-induced microglial cell activation. Mechanistic studies demonstrated that glycolysis inhibitors significantly inhibited LPS-induced phosphorylation of mechanistic target of rapamycin (mTOR), an inhibitor of nuclear factor-kappa B kinase subunit beta (IKKβ), and NF-kappa-B inhibitor alpha (IκB-α), degradation of IκBα, nuclear translocation of p65 subunit of NF-κB, and NF-κB transcriptional activity. In addition, 2-DG significantly inhibited LPS-induced acetylation of p65/RelA on lysine 310, which is mediated by NAD-dependent protein deacetylase sirtuin-1 (SIRT1) and is critical for NF-κB activation. A coculture study revealed that 2-DG reduced the cytotoxicity of activated microglia toward MES23.5 dopaminergic neuron cells with no direct protective effect. In an LPS-induced PD model, 2-DG significantly ameliorated neuroinflammation and subsequent tyrosine hydroxylase (TH)-positive cell loss. Furthermore, 2-DG also reduced dopaminergic cell death and microglial activation in the MPTP-induced PD model.

**Conclusions:**

Collectively, our results suggest that glycolysis is actively involved in microglial activation. Inhibition of glycolysis can ameliorate microglial activation-related neuroinflammatory diseases.

**Supplementary Information:**

The online version contains supplementary material available at 10.1186/s12974-021-02187-y.

## Introduction

Microglial cells are the resident immune effector cells of the central nervous system and are considered the cells critical for inflammation-associated neurotoxicit y[1]. Under physiological conditions, microglial cells play critical roles in maintaining brain homeostasis through surveillance of the microenvironment of the brain with ramified morphology [1]. Under neurodegenerative conditions or upon experimental stimulation with lipopolysaccharide (LPS) or interferon (IFN)-γ, microglial cells become activated with amoeboid morphology [2]. Once activated, microglial cells produce a number of proinflammatory cytokines and factors, such as tumor necrosis factor (TNF)-α, nitric oxide (NO), prostaglandin (PG)E_2_, interleukin (IL)-1β, IL-6, and reactive oxygen species (ROS), which in turn contribute to the recruitment of other immune cells and neuronal cell injuries [2, 3]. There is growing evidence that uncontrolled activation of microglial cells is involved in the progression of several neurodegenerative diseases, including Parkinson’s disease (PD) [4]. Neuroinflammation mediated by activated microglia was found in both PD patients and rodent models of PD and was positively associated with dopaminergic neuron loss [4]. Therefore, inhibition of microglial inflammatory activation has become a potential therapeutic strategy against PD.

Glucose metabolism is actively implicated in microglia/macrophage activation-mediated inflammatory responses [5, 6]. The glucose catabolic metabolism pathway consists of glycolysis and mitochondrial oxidative phosphorylation. Glycolysis is defined as the breakdown of glucose through a series of extramitochondrial biochemical reactions into pyruvic and lactic acids under anaerobic conditions. The process includes the glucose activation phase and the energy extraction phase [7]. Glycolytic flux is regulated by three rate-limiting steps that are mediated by three key enzymes: hexokinase (HK), phosphofructokinase (PFK), and pyruvate kinase (PK) [7]. In the last step of glycolysis, pyruvate is converted to lactate, which is catalyzed by dehydrogenase (LDH) under anaerobic conditions [8]. Several lines of evidence have suggested that LPS-activated M1 macrophage cells display metabolic reprogramming, switching from oxidative phosphorylation to aerobic glycolysis, whereas M2 (anti-inflammatory) macrophage/microglial cells switch to oxidative phosphorylation [9]. LPS stimulation elicits enhanced aerobic glycolysis of macrophages upon the switch from liver type-6-phosphofructo-2-kinase (PFK2) to the ubiquitous type-PFK2 expression [10]. Similar to macrophages or dendritic cells, activated M1-type microglial cells show a metabolic shift from oxidative phosphorylation to aerobic glycolysis [11].

Many studies have reported that inhibition of the glycolytic pathway can suppress inflammatory responses. Glucose transporter (Glut)1 was upregulated in activated microglial cells, and the inhibition of Glut-1 suppressed microglial activation [12]. Hexokinase (HK) 2 was induced in hypoxia-activated microglial cells, and the blockade of HK 2 suppressed ischemic brain injury by inhibiting microglia-mediated neuroinflammation [13]. 2-Deoxy-d-glucose (2-DG), a glucose analog, is phosphorylated by hexokinase and thereby competitively inhibits the production of glucose-6-phosphate from glucose and ultimately inhibits glycolysis [8]. In addition to its inhibitory effect on the proliferation of cancer cells, 2-DG has been recognized as a therapeutic agent for autoimmunity and inflammatory diseases [14]. The inhibition of glycolysis by 2-DG significantly reduced the production of some proinflammatory factors, such as high mobility group B (HMGB) and IL-1β, whereas it did not affect other proinflammatory cytokines, including TNF-α [15, 16]. However, in LPS-activated dendritic cells, 2-DG treatment substantially decreased the production of proinflammatory cytokines such as IL-6, IL-12, and TNF-α only at translational levels [17]. Although the neuroprotective effects of 2-DG in vitro and in vivo have been demonstrated [18–20], the anti-neuroinflammatory activity of glycolysis inhibition has rarely been reported. Recently, we found that 2-DG inhibited the expression of TNF-α, IL-6, iNOS, COX-2, and IL-1β at the transcriptional level [21]. However, the underlying mechanisms by which 2-DG acts to mitigate the transcriptional expression of proinflammatory genes, particularly in microglial cells, are unclear. Furthermore, the effects of 2-DG on inflammation-mediated dopaminergic neuron cell loss have not yet been investigated. In the present study, we addressed the role of aerobic glycolysis in LPS-activated microglial cells and its potential significance in inflammation-induced dopaminergic neuronal cell death.

## Methods and materials

### Materials

Bacterial lipopolysaccharide (LPS) (*Escherichia coli* serotype 055:B5), 1-methyl-4-phenyl-1,2,3,6-tetrahydropyridine (MPTP), 1-methyl-4-phenylpyridine(MPP^+^), 2-DG, and 3-bromopyruvic acid (3-BPA) were purchased from Sigma-Aldrich (St. Louis, MO, USA). Mouse TNF-α enzyme-linked immunosorbent assay (ELISA) kit and mouse IL-6 ELISA kit were obtained from R&D Systems (Minneapolis, MN). Antibodies used in this study were as follows: anti-phospho-inhibitor of nuclear factor kappa B kinase [p-IKKα (S176)/IKKβ (S177)], anti-p65 subunit of nuclear factor kappa B (NF-κB) (8242S), anti-NF-kappa B inhibitor alpha (IκBα) (4814S), anti-phospho-IκBα (S32) (2859S), anti-cyclooxygenase (COX)-2, anti-jun N-terminal kinase (JNK) (9252S), anti-phospho-JNK (T183/Y185) (4668S), anti-extracellular signal-regulated kinase (ERK) 1/2 (4695S), anti-phospho-ERK 1/2 (9102S), anti-p38 (9212S), anti-p-p38 (T180/Y182) (9215S), anti-phosho-p70-S6K (T421/S132) (9204S), anti-mechanistic target of rapamycin (mTOR) (2972S), anti-phospho-mTOR (S2448) (2971S), and anti-phospho-5′-AMP-activated protein kinase AMPK (T172) (2535S) antibodies were purchased from Cell Signaling Technology (Danvers, MA, USA); anti-inducible NO synthase (iNOS) (ab15323), anti-acetyl-p65 (acetyl K310) (ab19870), and anti-α-tubulin (ab7219) antibodies were from Abcam (Cambridge, MA, USA); and anti-hexokinase 2 (HK2) antibody was from Bioworld Technology (St. Louis Park, MN, USA). Anti-tyrosine hydroxylase (TH) (AB152) was obtained from Millipore (Billerica, MA, USA), and anti-ionized calcium-binding adaptor molecule 1 (Iba1) (019-19741) was purchased from Wako Chemicals (Chuo-ku, Osaka, Japan). Anti-CD68 (MCA 1957) was obtained from Bio-Red (*Hercules*, CA, *USA*).

### Cell culture

The BV-2 murine microglial cell line and human embryonic kidney (HEK) 293T cell line were cultured in Dulbecco’s modified Eagle’s medium (DMEM, Gibco, Carlsbad, USA) containing 10% fetal bovine serum (FBS, PAN Biotech and Lonsera, Aidenbach, Germany), 100 U/mL penicillin, and 100 μg/mL streptomycin (Gibco). MES23.5, a dopaminergic neuroblastoma cells, was derived from hybridization of rat embryonic mesencephalon cells and the murine neuroblastoma cell line N18TG2 [22]. The cell line was cultured in Dulbecco’s modified Eagle’s medium/nutrient mixture F-12 (DMEM/F-12, Gibco) with 5% fetal bovine serum (FBS, PAN Biotech, and Lonsera), 100 U/mL penicillin (Gibco), 100 μg/mL streptomycin (Gibco), and 100× insulin-transferrin-selenium (Gibco). All three cell lines were cultured in an incubator at 37°C in a 5% CO_2_ atmosphere.

### Primary culture of microglia

Primary microglial cells were collected from newborn C57BL/6J mice [23]. In summary, the newborn mice were washed in 75% alcohol, and the whole brains were isolated and minced in precooled PBS. Then, the cortical tissue was digested for 20 min with 0.25% trypsin. After centrifugation and resuspension, the samples were digested by DNaseI at 37°C and transferred to a single cell suspension. Then, single cells were plated on poly-d-lysine-coated flasks for 14 days. The microglial cells were obtained from mixed glial cultures on a shaker at 180 rpm for 3 h.

### Nitric oxide (NO) measurement

Microglial cells were seeded in a 96-well plate at a density of 2.0×10^4^ cells/well. After LPS stimulation for 24 h, 50 μl of cell culture supernatant was transferred to a new 96-well plate and mixed with 50 μl of Griess reagent. The signals were measured in a microplate reader (Infinite M200 PRO, Tecan, Switzerland) at 550 nm. The data were normalized to a standard curve.

### Cytotoxicity assay

Cell viability was measured by 3-(4,5-dimethylthiazol-2-yl)-2,5-diphenyltetrazolium bromide (MTT) assay. In brief, after appropriate treatment of BV-2 cells, 30 μl of MTT (Solarbio, Beijing, China) was added to each well and incubated at 37°C for 4 h. The samples were mixed with 100 μl of DMSO and detected at 540 nm by a microplate reader (Infinite M200 PRO).

### Enzyme-linked immunosorbent assay (ELISA)

The concentrations of TNF-α and IL-6 in the medium were detected by mouse TNF-α or IL-6 ELISA kits according to the manufacturers’ instructions.

### RNA isolation and quantitative real-time PCR

Total RNA from the microglial cells or tissues was isolated by TRIzol reagent and subjected to reverse transfection by Oligo-d(T) and M-MLV reverse transcriptase (Thermo Fisher, USA). Real-time quantitative PCR was performed on an Applied Biosystems 7500 Real-Time PCR system (Foster City, CA, USA) using PrimeScript RT Master Mix (TaKaRa, Dalian, China). The specific primers used in reverse transcription were purchased from GENEWIZ (Suzhou, China) as shown in Table 1. Gapdh was used as a control. The normalized CT values were calculated according to the comparative delta-delta Ct method.

**Table 1:** Primers used in real-time quantitative PCR

Primer	Sequence(5′–3′)	
mus-Gapdh	Forwad:	TGTGTCCGTCGTGGATCTGA
	Reverse:	TTGCTGTTGAAGTCGCAGGAG
mus-iNOS	Forwad:	TAGGCAGAGATTGGAGGCCTTG
	Reverse:	GGGTTGTTGCTGAACTTCCAGTC
mus-Cox-2	Forwad:	CAGGCTGAACTTCGAAACA
	Reverse:	GCTCACGAGGCCACTGATACCTA
mus-Tnf-α	Forwad:	CAGGAGGGAGAACAGAAACTCCA
	Reverse:	CCTGGTTGGCTGCTTGCTT
mus-Il-1β	Forwad:	TCCAGGATGAGGACATGAGCAC
	Reverse:	GAACGTCACACACCAGCAGGTTA
mus-Il-6	Forwad:	GCCAGAGTCCTTCAGAGAGA
	Reverse:	GGTCTTGGTCCTTAGCCACT
mus-Hk2	Forwad:	TCATTGTTGGCACTGGAAGC
	Reverse:	TTGCCAGGGTTGAGAGAGAG
mus-Glut-1	Forwad:	CAGTTCGGCTATAACACTGGTG
	Reverse:	GCCCCCGACAGAGAAGATG
mus-Ldha	Forwad:	TGTCTCCAGCAAAGACTACTGT
	Reverse:	GACTGTACTTGACAATGTTGGGA
mus-G6pdx	Forwad:	CACAGTGGACGACATCCGAAA
	Reverse:	AGCTACATAGGAATTACGGGCAA

### Western blot analysis

BV-2 microglial cells, HEK293T cells, and primary mixed glial cells were lysed on ice for 30 min and shaken every 10 min in denaturing lysis buffer (50 mmol/L Tris [pH 8], 1% Triton X-100, 0.1% SDS, 0.5% sodium deoxycholate, and 150 mmol/L NaCl and PMSF), while mouse tissue (the substantia nigra and the striatum) was measured by an ultrasound system before being lysed on ice. After mixing with 5×loading buffer, the proteins were separated by SDS-PAGE and transferred to a polyvinylidene difluoride (PVDF) membrane, followed by blocking in milk for 2 h. Then, the samples were incubated with the appropriate primary antibodies and HRP-conjugated secondary antibodies. Finally, proteins were detected by enhanced chemiluminescence (ECL) (Millipore) with a ChemiScope 3300 mini (CLINX, Shanghai, China).

### Plasmids and siRNA transfection

BV-2 microglial cells or primary mixed glial cells were seeded on a 12-well plate (5.0×10^4^ cells/well) 1 day prior to transfection. The cells were transfected with appropriate siRNA or plasmids accompanied by transfection reagents (Lipofectamine®RNAiMAX or Lipofectamine 2000) according to the manufacturer’s instructions. After 24 h, the cells were collected for subsequent experiments. The siRNA sequences are shown in Table 2.

**Table 2:** Sequences of si-RNA used in RNA interference

Gene	siRNA	Sense (5′–3′)
mus-Hk2	siHK2	GGAGAUGCGUAAUGUGGAATT
mus-Hk2 scramble		GUAAGGCGUAUGGAAUAGGTT
mus-Glut-1	siGLUT-1	GCUGCCUUGGAUGUCCUAUTT
mus-Glut-1 scramble		GUGUUACGUCUGUCGACUCTT
mus-Ldha	siLDHA	CCACCAUGAUUAAGGGUCUTT
mus-Ldha scramble		AGCUACGUCGAUUCUCAAGTT
Mus-Sirt1-1	siSIRT1-1	GCGCAUAGGUCCAUAUACUTT
Mus-Sirt1 scramble-1		GAUUUCCAUUCGCCAGTAGAT
Mus-Sirt1-2	siSIRT1-2	GCGCAUAGGUCCAUAUACUTT
Mus-Sirt1 scramble-2		GGUUUCUACAUCGUCCTACTA
Mus-Sirt1-3	siSIRT1-3	CCGUCUCUGUGUCACAAAUTT
Mus-Sirt1 scramble-3		GAUTUCCAUUCGCCAGUAGAT

### Coimmunoprecipitation (IP)

Protein was extracted in nondenaturing lysis buffer (20 mM Tris HCl [pH 8], 137 mM NaCl, 1% Nonidet P-40 (NP-40), and 2 mM EDTA), followed by incubation with appropriate antibodies overnight. Then, the samples were mixed with protein A/G beads for 4 h, and then, the beads were washed 10 times to remove nonspecifically bound proteins. The beads and protein were mixed with 2x loading buffer and separated in boiling water, followed by Western blotting.

### NF-κB luciferase reporter assays

BV-2 microglial cells or HEK293T cells stably expressing the NF-κB reporter were seeded on a 12-well plate one day before compound and LPS (200 ng/mL) stimulation. After 16 h, the cells were lysed in reporter lysis buffer, and luciferase activity was detected with a dual-luciferase assay kit following the manufacturer’s protocol (Promega, USA).

### MPTP-induced PD model

Mice (male, C57BL/6J, 6–8 weeks) were obtained from the Shanghai SLAC Laboratory Animal Co., Ltd. (Shanghai, China) and placed in an SPF laboratory animal room. All the experiments used in this research followed the Guide for the Care and Use of Laboratory Animals (8th edition) and were approved by the Institutional Animal Care and Use Committee of Soochow University. In this experiment, mice were randomly allocated to 3 groups: saline, MPTP alone (30 mg/kg), and 2-DG (400 mg/kg) +MPTP (30 mg/kg). For the 2DG+MPTP group, 2-DG was given 3 days prior to MPTP injections. MPTP was injected for 7 consecutive days, and 2-DG was preadministered 2 h before MPTP injection. The rotarod test and pole test were performed on the 11th day. After behavioral tests, mouse brain tissue was collected for further study. All behavioral tests were performed by an investigator blind to the treatment.

### LPS-induced PD model

Mice (male, C57BL/6J, 6–8 weeks) were randomly separated into 3 groups: saline, LPS alone (5 mg/kg), and 2-DG (400 mg/kg) +LPS (5 mg/kg). For the 2DG+LPS group, 2-DG was given 3 days prior to LPS injections. LPS was stereotactically injected into the substantia nigra pars compacta (SNc) on the third day, and 2-DG was administered for 7 consecutive days. On the 11th day, the mice were euthanized, and brain tissue was collected for further study.

### Rotarod test

Before the test, mice were trained on the rotarod for 10 min. During testing, each mouse was placed on the rotarod with increasing speed from 4 rpm/min to 40 rpm/min, and the time when mice fell from the rotarod was recorded. The test for each mouse was recorded 3 times. The average of three trials was used as a statistical indicator [24]. The examiner conducting the rotarod test was blind to the treatment.

### Pole test

The pole used in this experiment is a wooden stick with a wooden ball on the top that has a rough surface. During testing, mice were placed on the wooden ball, and the time for the mice to climb from the ball to the base of the stick was recorded. The test for each mouse was recorded 3 times. The average of three trials was used as a statistical indicator. The examiner conducting the pole test was blind to the treatment.

### Immunohistochemistry

The mice were euthanized, and normal saline was perfused into the left ventricle, followed by paraformaldehyde (4%) perfusion. After perfusion, the mouse brains were collected and fixed in phosphate-buffered paraformaldehyde for 3 days at 4°C and then dehydrated in 30% sucrose solutions until they sank. The brain was frozen and cut into 20-μm slices with a freezing microtome. For immunofluorescence staining, the brain slices were washed in PBS and blocked with fetal bovine serum (FBS). Then, the samples were soaked with the appropriate antibody overnight. The next day, brain slices were washed in PBST for 30 min before or after incubation with secondary fluorescent antibodies for 2 h in dark. The samples were placed on a microscope slide and observed by confocal microscopy (Zeiss LSM710 META, Germany).

For histological quantification, every fifth 20-μm-thick section of the region spanning bregma −2.92 to −3.64 mm was analyzed. Five sections of clear Substantia Nigra region per mouse were first delineated using a 4× objective. The number of TH positive cells was quantified manually using 10× objective. It ensured that we selected the same regions of interest among the different groups, and three mice of each group were used for statistical analysis. Similarly, the optical density (OD) of Iba1or CD68 immunoreactivity in the same sections was measured using ImageJ analysis software with 10× objective. Scorings of TH-positive cells and optical density of Iba1 or CD68 immunostaining were performed by a researcher blind to experiment treatment.

### ADP/ATP ratio assay

An ADP/ATP assay kit (Sigma-Aldrich) was used in the ADP/ATP ratio assay. In brief, BV-2 cells were seeded in a 12-well plate (1×10^5^ cells/well). After LPS stimulation for 16 h, the cells were digested and collected in a centrifuge tube. Then, the cells were seeded in a 96-well plate with a white background (1×10^4^ cells/well). The ATP reagent was prepared and added to each well. The samples were placed at room temperature for 1 min and measured by a luminescence reporter assay system (Promega, Madison, WI, USA). The value was recorded as [(RLU)A]. After 10 min, the samples were measured again as [(RLU)B]. After recording [(RLU)B], 5 mL of ADP reagent was prepared and added to samples without delay. Then, the samples were incubated at room temperature for 1 min before the luminescence was detected as [(RLU)C]. The ADP/ATP ratio was calculated following the manufacturer’s instructions.

### NAD^+^/NADH assay

After LPS stimulation for 16 h, BV-2 microglial cells were digested and resuspended into two precooled tubes. Then, 100 μL of NAD extraction buffer or NADH extraction buffer was prepared and added to each tube. After heating at 60°C for 5 min, 20 μL of assay buffer and 100 μL of the opposite extraction buffer were added to each sample, followed by centrifugation at 14,000 rpm for 5 min. Finally, 80 μL of working reagent was added to each sample, and the optical density was detected immediately (OD_0_) and 15 min later (OD_15_). NAD+/NADH was calculated according to the manufacturer’s instructions.

### Extracellular flux assays

Extracellular flux assays (Seahorse Bioscience, Chicopee, MA) were used to measure the oxygen consumption rate (OCR) and extracellular acidification rate (ECAR) of BV-2 microglial cells. BV-2 microglial cells were plated in a Seahorse XF microplate at a density of 2.0×10^4^ cells/well and cultured overnight. Then, the plate was balanced in a non-CO_2_ incubator at 37°C for 30 min. Meanwhile, Seahorse XF Glycolysis Stress Test Kit assay medium was prepared, and glutamine was added to Seahorse XF Base Medium. Finally, the compounds were diluted in an assay medium and added to the microplate. The ECAR and OCR were measured in an Agilent Seahorse XFe/XF96 or 24 Analyzer.

### Coculture

Microglia cells were seeded in triplicate at a density of 5x10^4^ in a 24-well plate. Microglia cells were pretreated with 2-DG for 30 min before LPS addition. After stimulation with LPS for 6h, the cell culture supernatant was discarded and fresh media were added. After cultured for 24h, the condition media (CM) from compound or vehicle-treated microglia cells were collected. MES23.5 dopaminergic neural cells were cultured in a 96-well plate (2.0×10^4^ cells/well), and after 12 h, the conditioned medium was added to MES23.5 for 24 h. Then, cell viability was measured by MTT assay and flow cytometry.

### Flow cytometry

BV-2 microglial cells were treated with 2-DG for 30 min prior to lipopolysaccharide (LPS)-Alexa Fluor® 488 (L23351, Invitrogen, CA, USA) stimulation. After 2 h, cells were resuspended and washed in PBS for 3 times. The cell-associated fluorescence was measured by flow cytometry analysis (Beckman Coulter, Brea, CA, USA).

### Statistical analysis

All data are presented as the mean ± standard deviation (S.D.) from at least three independent experiments and analyzed with GraphPad Prism software (version 8.0.1; San Diego, CA, USA). Comparisons between multiple groups were analyzed by one-way analysis of variance (ANOVA) or two-way analysis of variance (ANOVA) with Tukey’s test. *p*< 0.05 was considered statistically significant.

## Results

### LPS rapidly induces microglial aerobic glycolysis

**Fig. 1:**
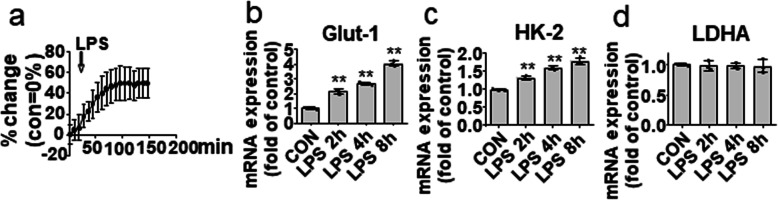
LPS rapidly induces microglial aerobic glycolysis. **a** Real-time changes in the ECAR of the BV-2 microglial cells treated with LPS (200 ng/mL) (arrow indicates initiation time of LPS treatment). BV-2 microglial cells were treated with LPS for the indicated times, and then, the expression of Glut-1 (**b**), HK-2 (**c**), and LDHA (**d**) was determined by quantitative real-time PCR (Q-PCR). Data are presented as mean ± S.D. (n=3) and are representative of results obtained from three independent experiments. *p<0.05, **p<0.01, compared to the control group

The agonists of Toll-like receptors lead to a rapid increase in the glycolytic rate in dendritic cells [17]. We therefore determined whether LPS can also trigger aerobic glycolysis in microglial cells within a short period of stimulation. The results showed that the glycolytic rate (ECAR) was indeed upregulated within 150 min of LPS treatment (Fig. 1a). We then evaluated the expression of glycolytic genes, including Glut-1, HK2, and lactate dehydrogenase A (LDHA). Among these, the expression of Glut-1 and HK2 was upregulated, while the expression of LDHA was not affected in the LPS-activated BV-2 microglial cells (Fig. 1b–d). These results indicate that LPS can rapidly induce an increase in aerobic glycolysis in microglial cells.

### Aerobic glycolysis is required for microglial inflammatory activation

**Fig. 2:**
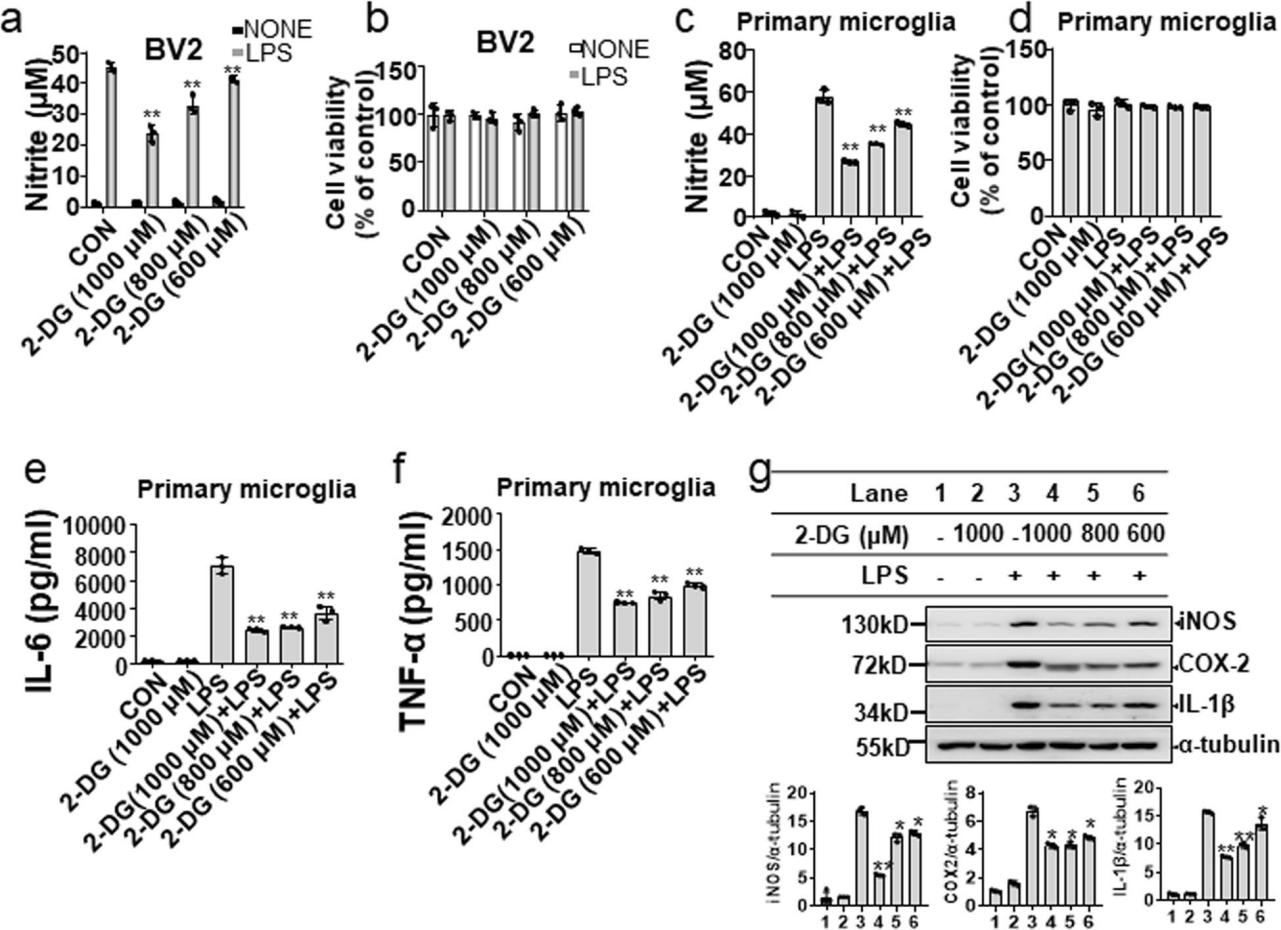
Aerobic glycolysis is required for microglial inflammatory activation. BV-2 microglial cells or primary microglial cells were pretreated with 2-DG (600-1000 μM) for 30 min, followed by LPS (200 ng/mL) treatment for 24 h. The nitrite in the cell culture medium was determined using the Griess reaction (**a** BV-2 microglial cells; **c** primary microglia). Cell viability was determined by MTT assay (**b** BV-2 microglial cells; **d** primary microglia). The concentrations of IL-6 (**e**) and TNF-α (**f**) in the primary microglial cell culture media were analyzed by ELISAs. The expression of iNOS, COX-2, and IL-1β (**g**) in BV-2 microglial cells was determined by Western blotting (*upper*), and the relative protein levels were quantified by densitometry analysis (*lower*). Data are presented as mean ± S.D. (n=3) and are representative of results obtained from three independent experiments. *p < 0.05, **p <0.01, compared to the NONE group or LPS alone group

To test whether glycolysis is required for microglial activation, we employed a small-molecule inhibitor or siRNA against glycolytic genes to interfere with glucose metabolism and measured proinflammatory gene expression as a functional output. Glycolytic inhibitors (2-DG and 3-BPA) significantly inhibited LPS-induced production of nitric oxide (NO) in a dose-dependent manner in BV-2 microglial cells as well as primary microglial cells (Fig. 2a, c; Fig. s1a and s1c), while at the tested concentrations, these inhibitors did not affect microglial cell viability (Fig. 2b, d; Fig. s1b and s1d). The levels of proinflammatory cytokines including TNF-α and IL-6 in cell culture supernatants were measured by ELISA. The results showed that glycolytic inhibitors significantly reduced LPS-induced increase of TNF-α and IL-6 in BV-2 microglial cells (Fig. s1e-h). The effects of 2-DG on LPS-induced of TNF-α and IL-6 were further confirmed in primary microglia cultures (Fig. 2e, f). The results showed that 2-DG significantly reduced TNF-α and IL-6 production in LPS-activated primary microglial cells, indicating that inhibitory effects of 2-DG on the production of proinflammatory factors are not limited to BV-2 microglial cells. The expression of IL-1β, iNOS, and COX-2 was determined by Western blotting. Pretreatment with glycolytic inhibitors suppressed expression of this protein expression in LPS-activated BV-2 microglial cells (Fig. 2g; Fig. s1i). The effects of 2-DG on the expression of COX-2 and IL-1β were also confirmed in LPS-primary mixed glial cells. 2-DG decreased LPS-induced expression of COX-2 and IL-1β in primary mixed glial cells (Fig. s1j). Importantly, glycolytic inhibitors also inhibited proinflammatory genes at the transcriptional level in the LPS-activated BV-2 microglial cells (Fig. s2). We next examined whether the knockdown of glycolytic genes can inhibit proinflammatory gene expression. As shown in Fig. s3, knockdown of Glut-1, HK2, or LDHA significantly suppressed LPS-induced expression of TNF-α, IL-6, iNOS, COX-2, and IL-1β at the transcriptional level in the BV-2 microglial cells. These results suggest that inhibition of glycolysis reduces the expression of proinflammatory genes at both protein and mRNA levels.

### Glycolytic inhibitors suppress LPS-induced activation of NF-κB signaling pathway

**Fig. 3:**
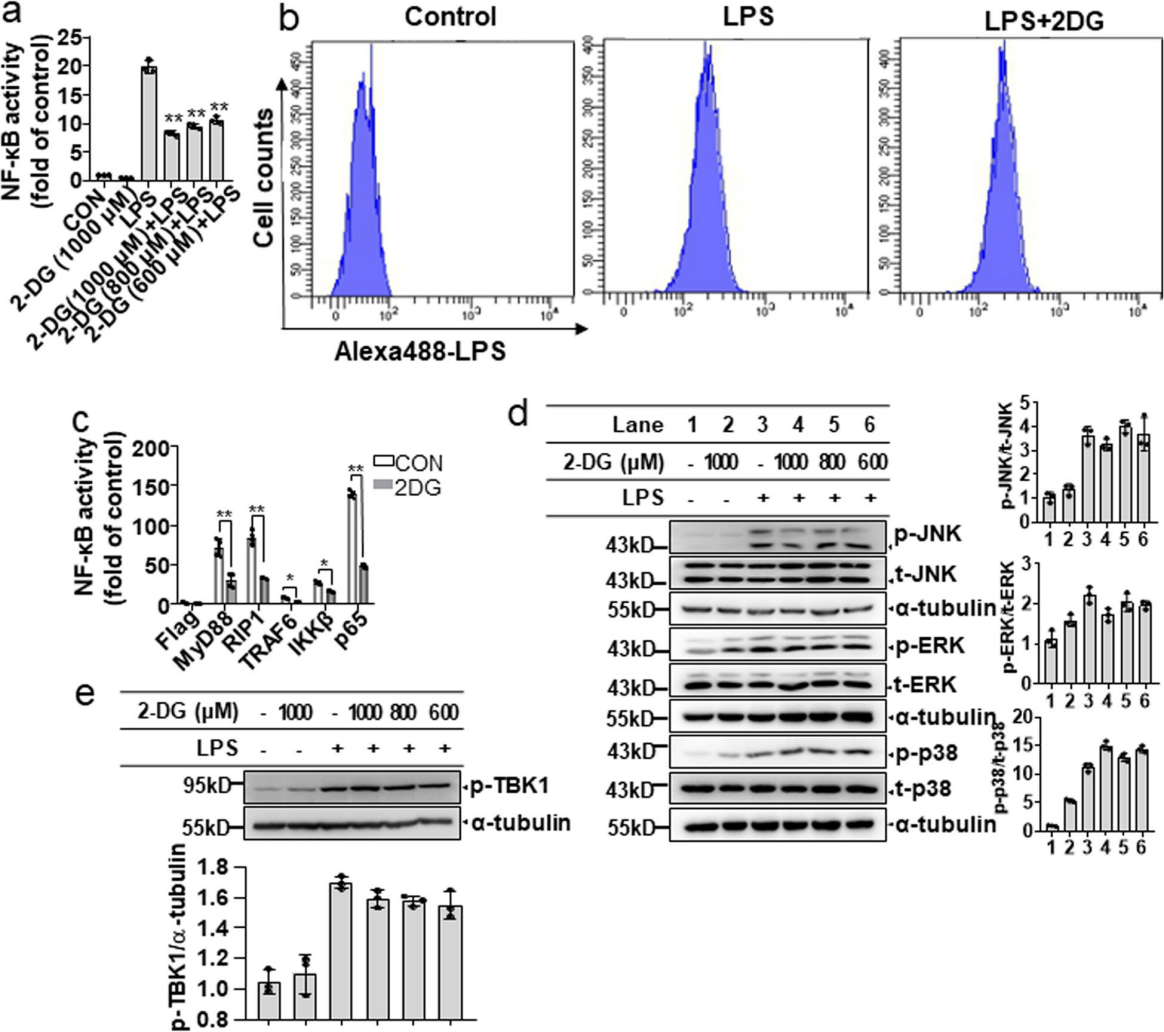
Glycolytic inhibitors suppress the inflammatory response by negatively regulating NF-κB signaling pathways. **a** BV-2 microglial cells stably expressing NF-κB luciferase reporter construct were pretreated with 2-DG (600–1000 μM) for 30 min, followed by LPS (200 ng/mL) stimulation for 16 h. The transcriptional activity of NF-κB was determined by luciferase reporter assay. **b** BV-2 microglial cells were pretreated with 2-DG for 30 min, followed by treatment of lipopolysaccharide (LPS)-Alexa Fluor® 488 for 2 h. The binding efficiency was analyzed using flow cytometry. **c** HEK293T cells stably expressing NF-κB luciferase reporter construct were transfected with indicated plasmid. After a 16-h transfection, the cells were treated with 2-DG for 8 h. The NF-κB transcriptional activity was determined by luciferase reporter assay. **d** BV-2 microglial cells were pretreated with 2-DG (1000 μM) for 30 min, followed by LPS (200 ng/mL) treatment for 30 min. The expression of p-JNK, JNK, p-ERK, ERK, p-p38, and p38 was detected by Western blotting (*left*), and the relative protein levels were quantified by densitometric analysis (*right*). **e** BV-2 microglial cells were treated with 2-DG (1000 μM) for 30 min prior to LPS stimulation for 30 min. The expression of p-TBK1 was analyzed by Western blotting (*upper*), and the relative protein levels were quantified by densitometric analysis (*lower*). Data are presented as mean ± S.D. (n=3) and are representative of results obtained from three independent experiments. *p < 0.05, **p <0.01 compared to the LPS alone group or control group

Transcriptional factor NF-κB is a key regulator of the transcriptional expression of proinflammatory factors in LPS-activated microglial cells [25]. We therefore examined whether NF-κB is involved in the inhibitory effects of glycolytic inhibitors on proinflammatory gene expression. The NF-κB luciferase activity results showed that both 2-DG and 3-BPA dose-dependently suppressed LPS-induced NF-κB transcriptional activation in BV-2 microglial cells (Fig. 3a; Fig. s4a). To further investigate the inhibitory mechanisms of glycolytic inhibitors on the NF-κB signaling pathway, the upstream signaling pathways were evaluated. We first examined the effects of glycolytic inhibitors on the binding of Alexa488-conjugated LPS to BV-2 microglial cells by flow cytometry analysis. The results showed that 2-DG did not affect FITC-LPS binding to the cell surface membranes of the BV-2 microglial cells (Fig. 3b). Next, we transfected various upstream molecules of the NF-κB signaling pathway, including myeloid differentiation primary response 88 (MyD88), receptor-interacting serine/threonine-protein kinase 1 (RIP1), TNF receptor associated factor 6 (TRAF6), IKKβ, and p65 into HEK293T cells stably expressing the NF-κB luciferase reporter construct and evaluated the effects of glycolytic inhibitors on the induced NF-κB luciferase activity. As shown in Fig. 3c and Fig. s4b, overexpression of MyD88, RIP1, TRAF6, IKKβ, and p65 strongly induced NF-κB luciferase activity in the HEK293T cells, and both 2-DG and 3-BPA significantly suppressed the induced NF-κB luciferase activity. The kinase TAK1 not only phosphorylates IKKβ, but also activates mitogen-activated protein kinases (MAPKs), resulting in the activation of activating protein-1 (AP-1), which is another key transcriptional factor for the expression of proinflammatory genes in LPS-activated microglial cell s[26]. We thus investigated the effects of glycolytic inhibitors on MAPK activation. As shown in Fig. 3d and Fig. s4c, neither 2-DG nor 3-BPA suppressed the phosphorylation of JNK, ERK, and p38 induced by LPS. It was reported that TBK-IKKε signaling plays crucial roles in TLR-derived early glycolytic reprogramming in dendritic cells. Therefore, we sought to determine whether glycolytic inhibitors suppress TBK1 phosphorylation in LPS-activated BV-2 microglial cells. The results showed that LPS-induced TBK1 phosphorylation was not affected by glycolytic inhibitors (Fig. 3e; Fig s4d). Taken together, these results indicate that downstream of TAK1, IKKβ, and p65 may be targets of glycolytic inhibitors in LPS-driven NF-κB activation.

### Glycolytic inhibitors suppress LPS-induced IKKβ activation by modulating AMPK/mTOR signaling in microglial cells

**Fig. 4:**
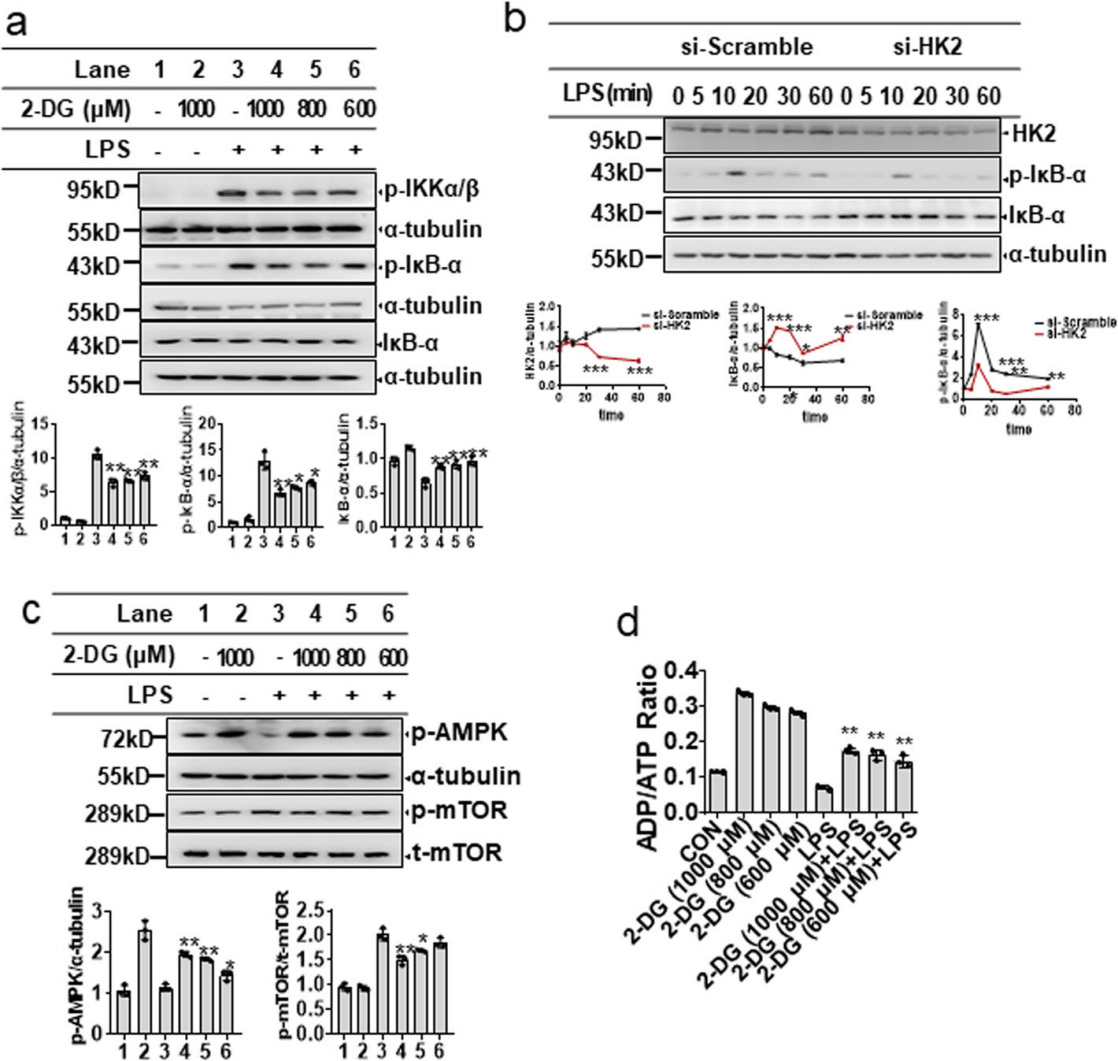
Glycolytic inhibitors suppress LPS-induced IKKβ activation by modulating AMPK/mTOR signaling in microglial cells. **a** BV-2 microglial cells were pretreated with 2-DG (600–1000 μM) for 30 min, followed by LPS (200 ng/mL) treatment for 15 min. The expression of p-IKKα/β**,** p-IκB-α, and IκB-α was analyzed by Western blotting (*upper*), and the relative protein levels were quantified by densitometric analysis (*lower*). **b** BV-2 microglial cells were transfected with si-Scramble or si-HK2. After 48 h, the cells were stimulated with LPS (200 ng/ml) for the indicated time (0, 5, 10, 20, 30, 60 min), and then, the expression of HK2, p-IκB-α, and IκB-α was determined by Western blotting (*upper*), and the relative protein levels were quantified by densitometric analysis (*lower*). **c** BV-2 microglial cells were pretreated with 2-DG (600–1000 μM) for 30 min, followed by LPS (200 ng/mL) treatment for the indicated time. After 1 h, the expression of p-AMPK and p-mTOR was determined by Western blotting (*upper*), and the relative protein levels were quantified by densitometric analysis (*lower*). **d** BV-2 microglial cells were pretreated with 2-DG (600–1000 μM) for 30 min, followed by LPS (200 ng/mL) treatment for 16 h. The ADP/ATP ratio was calculated as described in the methods and materials section. Data are presented as mean ± S.D. (n=3) and are representative of results obtained from three independent experiments. *p < 0.05, **p <0.01, compared to the LPS group

Given that IKKβ is a major downstream signaling molecule in TLR4-mediated inflammatory responses, we next sought to determine whether glycolytic inhibitors can suppress LPS-induced IKKβ activation. The results revealed that both 2-DG and 3-BPA inhibited LPS-induced phosphorylation of IKKα/β in BV-2 microglial cells in a dose-dependent manner (Fig. 4a; Fig. s5a). Since the activation of IKKβ induces phosphorylation and degradation of IκBα, we explored the effect of glycolytic inhibitors on phosphorylation and degradation of IκBα. As shown in Fig. 4a and Fig. s5a, both 2-DG and 3-BPA reduced the phosphorylation and degradation of IκBα in BV-2 microglial cells in a dose-dependent manner. The effects of 2-DG on activation of IKKβ and degradation of IκBα were confirmed in primary mixed glial cells. The results revealed that 2-DG inhibited LPS-induced phosphorylation of IKKβ and degradation of IκBα in primary mixed glial cells (Fig. s5b). We also observed that knockdown of HK2 inhibited LPS-induced phosphorylation and the subsequent degradation of IκBα in BV-2 microglial cells or mixed primary glial cells (Fig. 4b; Fig. s5c). These results indicate that the anti-inflammatory effects of glycolytic inhibitors may be related to IKKβ inhibition.

AMPK/mTOR has been reported to positively regulate IKKβ activation [27, 28]. Therefore, we investigated the effects of glycolytic inhibitors on AMPK phosphorylation and mTOR phosphorylation in LPS-activated BV-2 microglial cells. Both 2-DG and 3-BPA decreased the phosphorylation of mTOR and increased the phosphorylation of AMPK in the LPS-activated BV-2 microglial cells (Fig. 4c; Fig. s5d). The effects of 2-DG on phosphorylation of AMPK and mTOR were further confirmed in primary mixed glial cells. The results showed that 2-DG decrease LPS-induced the phosphorylation of mTOR and increased the phosphorylation of AMPK in primary mixed glial cells (Fig. s5e), indicating that the effects of 2-DG on AMPK/mTOR signaling is not limited to BV-2 microglial cells. Since AMPK is directly regulated by the ADP/ATP ratio, we also measured the ADP/ATP ratio in the BV-2 microglial cells. 2-DG and 3-BPA increased the ADP/ATP ratio both in the activated and inactivated BV-2 microglial cells, corresponding to AMPK phosphorylation (Fig. 4d; Fig. s5f). These results revealed that glycolytic inhibitors suppressed IKK phosphorylation by increasing AMPK phosphorylation and inhibiting mTOR phosphorylation.

### Glycolytic inhibitors suppress LPS-induced transcriptional activity of NF-κB via inhibition of NAD^+^/SIRT1 mediated p65 acetylation

**Fig. 5:**
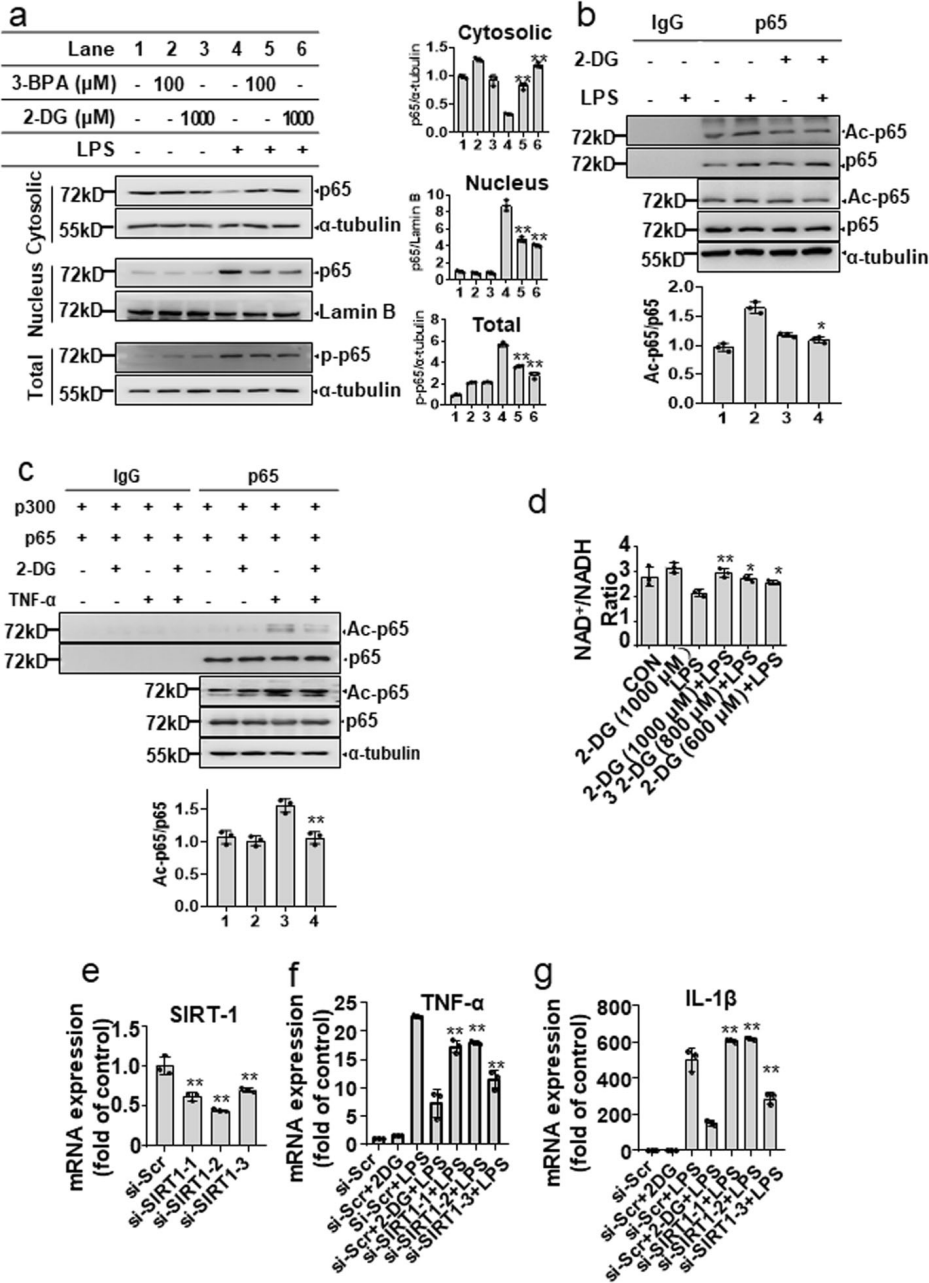
Glycolytic inhibitors suppress LPS-induced transcriptional activity of NF-κB via inhibition of NAD+/SIRT1-mediated p65 acetylation. **a** BV-2 microglial cells were pretreated with 2-DG (1000 μM) or 3-BPA (100 μM) for 30 min, followed by LPS (200 ng/mL) treatment for 1 h. The expression of p65 subunit of NF-κB p65 and p-p65 subunit of NF-κB in the total, cytosolic, and nucleus lysates was determined by Western blotting (*left*), and the relative protein levels were quantified by densitometric analysis (*right*). **b** BV-2 microglial cells were pretreated with 2-DG (1000 μM) for 30 min followed by LPS (200 ng/mL) treatment for 1 h. The extracts were subjected to immunoprecipitation (IP) with anti-p65 antibody or anti-IgG followed by immunoblotting analysis with anti-acetylated-p65 antibody and anti-p65 antibody. The expression of ac-p65 was determined by Western blotting (*upper*), and the relative protein levels were quantified by densitometric analysis (*lower*). **c** HEK293T cells were cotransfected with p300 and p65 plasmids for 24 h, followed by 2-DG (1000 μM) treatment for 30 min and then treated with TNF-α (10 ng/mL) for 1 h. The extracts were subjected to immunoprecipitation (IP) with anti-p65 or anti-IgG followed by immunoblotting analysis with anti-acetylated-p65 antibody and anti-p65 antibody. The expression of ac-p65 was determined by western blotting (*upper*) and the relative protein levels were quantified by densitometric analysis (*lower*). **d** BV-2 microglial cells were pretreated with 2-DG (600–1000 μM) for 30 min, followed by LPS (200 ng/mL) treatment for 16 h. The NAD^+^/NADH ratio was measured as described in the methods and materials section. **e–g** BV-2 microglial cells were transfected with si-SIRT1 (40 nM) or si-Scramble for 48 h, followed by LPS (200 ng/mL) treatment for 6 h. The mRNA expression of SIRT1, TNF-α, and IL-1β was measured by qPCR. Data are presented as mean ± S.D. (n=3) and are representative of results obtained from three independent experiments. *p<0.05, **p <0.01 compared to the LPS, si-Scramble or si-Scramble + LPS group, respectively

Since glycolytic inhibitors reduced p65 overexpression-induced NF-κB transcriptional activation, we tested the effects of glycolytic inhibitors on p65 nuclear translocation. The results of the nucleus-cytoplasm fraction assay revealed that both 2-DG and 3-BPA markedly inhibited LPS-induced nuclear distribution of the p65 subunit of NF-κB in BV-2 microglial cells (Fig. 5a). The effects of 2-DG on the nuclear distribution of the p65 subunit of NF-κB were also confirmed in primary microglial cells and mixed glial cells. 2-DG markedly suppressed p65 nuclear translocation in LSP-activated primary microglial cells and mixed glial cells, similar to in BV-2 microglial cells (Fig. s6). The acetylation of p65 at lysine 310 was demonstrated to promote the transcriptional activity of NF-κB [29]. Therefore, we asked whether a glycolytic inhibitor can decrease p65 acetylation levels at lysine 310 in LPS-activated BV-2 microglial cells. The results showed that LPS increased the acetylation of p65 levels and that 2-DG significantly decreased LPS-induced p65 acetylation (Fig. 5b). This finding was confirmed in the TNF-α-treated HEK 293T cells that were cotransfected with p300 and the p65 subunit of NF-κB (Fig. 5c). Acetylation of p65 is suppressed by Sirtuin 1 (SIRT1), whose activity is dependent on intracellular NAD^+^ levels, which are regulated by glucose metabolism [30, 31]. We therefore tested whether the inhibitory effects of 2-DG on NF-κB activation were dependent on SIRT1 activation. As shown in Fig. 5d, 2-DG rescued the LPS-induced decrease in the NAD^+^/NADH ratio in BV-2 microglial cells. Knockdown of SIRT1 partly blocked the inhibitory effect of 2-DG on inflammatory cytokine production (IL-1β and TNF-α) in LPS-activated BV-2 microglial cells (Fig. 5e, g). These results indicate that the reduction of NAD^+^-mediated p65 acetylation is involved in the anti-inflammatory activity of glycolysis inhibition.

### Neuroprotective effects of glycolytic inhibitors in activated microglial CM/neuron coculture model

**Fig. 6:**
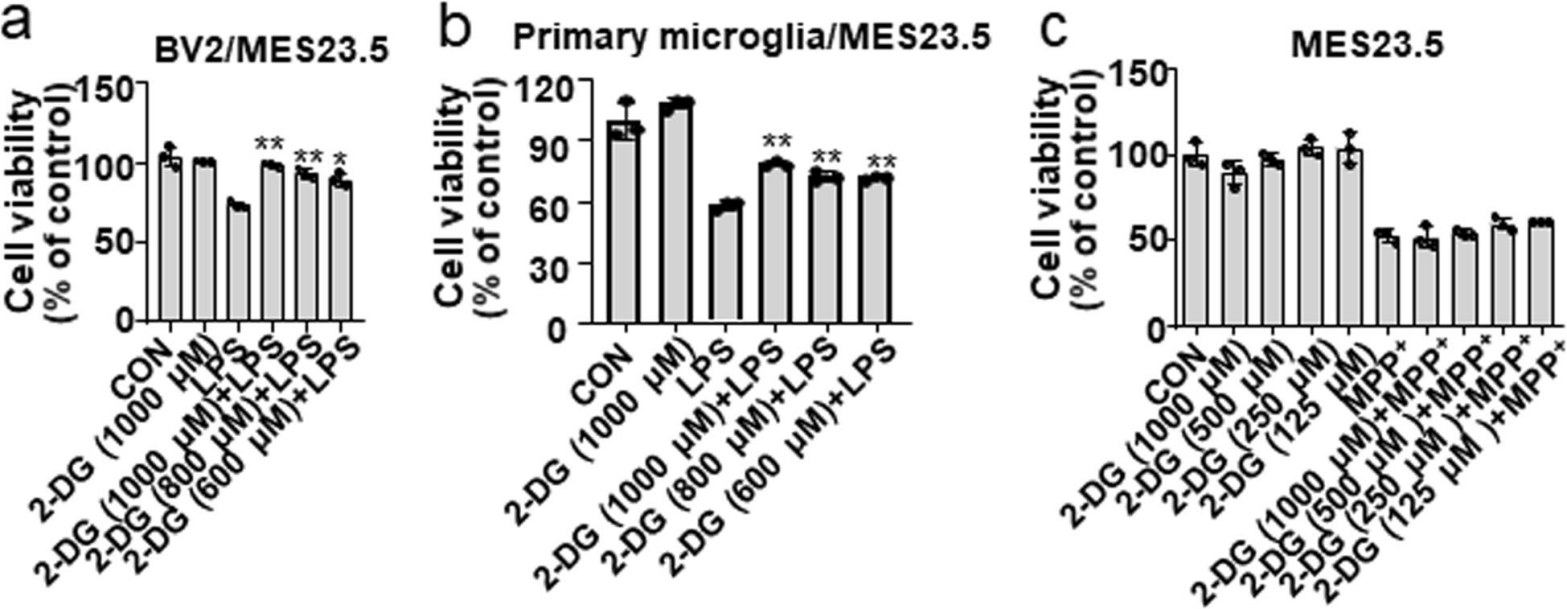
Glycolytic inhibitors reduce CM of activated microglial cell-induced MES23.5 neuroblastoma cell death. **a** BV-2 microglial cells were pretreated with 2-DG (600–1000 μM) for 30 min, followed by LPS (200 ng/mL) treatment for 6 h. The culture media was removed, and fresh medium was added. After 24 h of incubation, culture medium was added to the MES23.5 cells. MES23.5 cells were incubated with CM for 24 hours, and then, the cell viability was accessed by MTT assay. Cell viability was determined by MTT assay. **b** Primary microglial cells were pretreated with 2-DG (600–1000 μM) for 30 min, followed by LPS (200 ng/mL) treatment for 6 h. The culture media were removed, and fresh medium was added. After 24 h of incubation, culture medium was added to the MES23.5 cells. MES23.5 cells were incubated with CM for 24 h, and then, the cell viability was accessed by MTT assay. **c** MES23.5 cells were pretreated with 2-DG (125–1000 μM) before stimulation of MPP^+^ (1000 μM) for 24 h. Cell viability was determined by MTT assay. Data are presented as mean ± S.D. (n=3) and are representative of results obtained from three independent experiments. *p <0.05, **p <0.01, compared to the LPS or MPP^+^-alone group

Several lines of evidence suggest that proinflammatory factors derived from overactivated microglial cells can induce injury to or death of surrounding dopaminergic neuronal cell in vitro and in vivo [2]. Since glycolytic inhibitors exhibited anti-inflammatory activity, we speculated that the inhibition of glycolysis might mitigate microglial activation-induced dopaminergic neuronal cell death. To examine this possibility, a BV-2 microglial cell-conditioned media (CM)/MES23.5 dopaminergic neuronal cell coculture system was employed [32]. As shown in Fig. 6a and Fig. s7a, while MES23.5 cells were incubated with CM from LPS-stimulated BV-2 microglial cells, the cell viability was significantly decreased, and pretreatment with glycolytic inhibitors significantly attenuated this effect. The neuroprotective effect of 2-DG was also confirmed in a primary microglial cell-CM/MES23.5 cell coculture system. The viability of MES23.5 cells being treated with CM collect from 2-DG pretreated primary microglial cells was significantly improved (Fig. 6b). It has been reported that 2-DG attenuated rotenone- or Fe^2+^-induced SK-N-MC neuroblastoma cell death [20]. We thus examined whether glycolysis directly ameliorated the MPP^+^-induced reduction in MES23.5 cell viability. The results showed that neither 2-DG nor 3-BPA at the tested concentrations blocked MPP^+^-induced MES23.5 cell death (Fig. 6c; Fig. s7b). These results indicated that the inhibition of glycolysis is neuroprotective, which is likely attributable to the suppression of microglial inflammatory activation.

### Glycolytic inhibitor 2-DG ameliorates neuroinflammation and inflammation-mediated dopaminergic cell loss in an LPS-induced mouse PD model

**Fig. 7:**
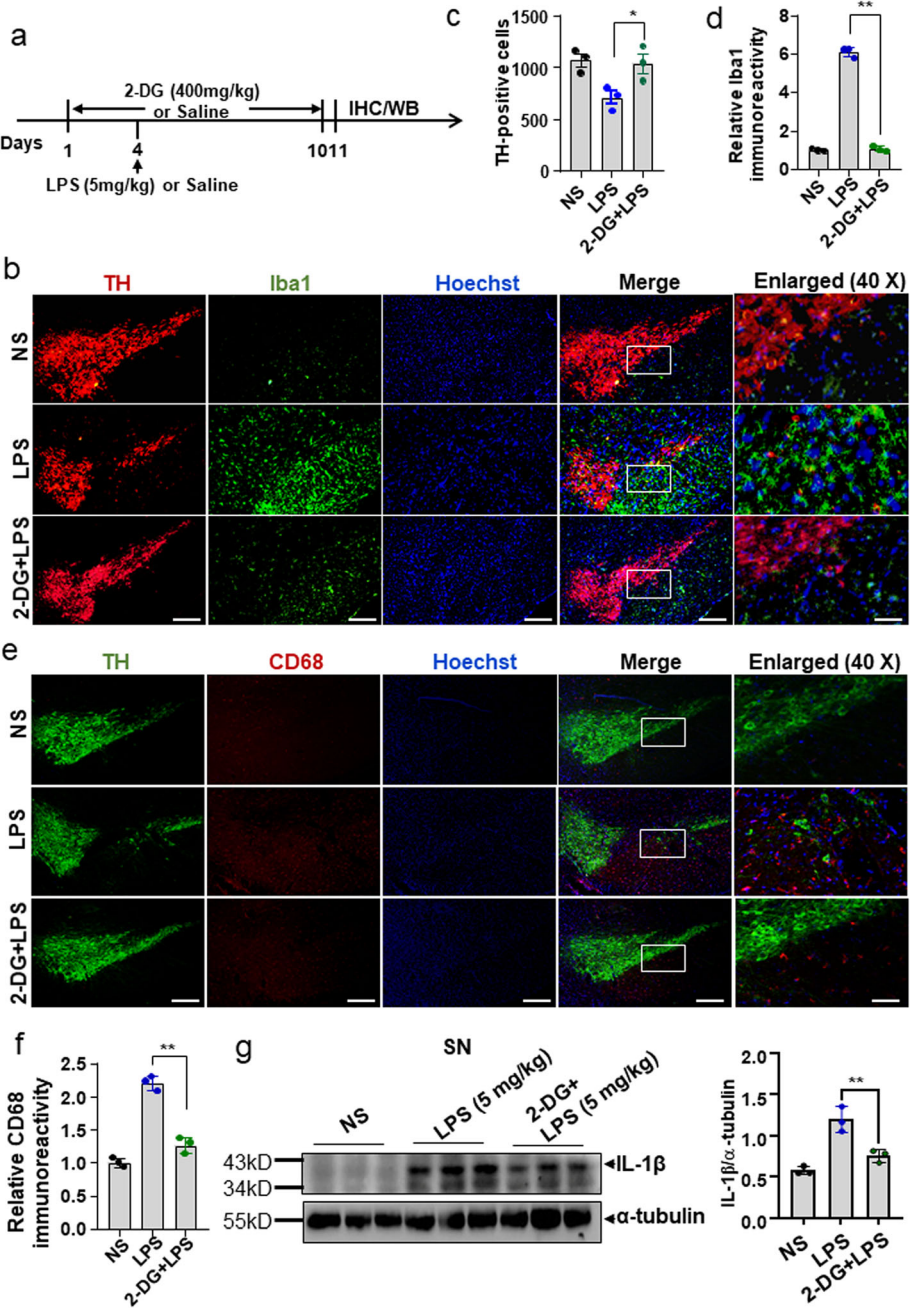
Glycolytic inhibitor 2-DG ameliorates neuroinflammation and inflammation-mediated dopaminergic cell loss in an LPS-induced mouse PD model. **a** Schematic diagram of drug administration. Mice were injected intraperitoneally with 2-DG (400 mg/kg/day) or saline (NS) for sequential 10 days. On the fourth day, mice were injected stereotactically with LPS (5 mg/kg) or saline (NS). After 10 days, the mice were euthanized for IHC analysis. **b** Double immunostaining for TH and Iba1 in the SN. **c**, **d** The number of TH positive cells was quantified manually, and the optical density of Iba1 immunoreactivity in same sections was measured using ImageJ analysis software (n=3 mice per group). Magnification, ×10, scale bar, 100 μm (*left*). Boxed rectangular regions were enlarged (*right*). Magnification, ×40, scale bar, 25 μm. **e** Double immunostaining for TH and CD68 in the SN. **f** The optical density of CD 68 immunoreactivity was measured using ImageJ analysis software (n=3 mice per group). Magnification, ×10, scale bar, 100 μm. Boxed rectangular regions were enlarged (*right*). Magnification, ×40, scale bar, 25 μm. Data are presented as the means ± S.E.M. *p <0.05, **p <0.01, compared to the LPS group. **g** The expression IL-1β in the SN was determined by Western blotting (*left*), and the relative protein levels were quantified by densitometric analysis (*right,* n=3 mice per group). Data are presented as the means ± S.D. *p <0.05, **p <0.01, compared to the LPS group

Given the anti-inflammatory and subsequent neuroprotective effects of the glycolytic inhibitors in vitro, we further assessed the inhibitory effects on neuroinflammation in an intracranial LPS-induced mouse PD model (Fig. 7a). Mice were administered 2-DG (400 mg/kg once a day) by intraperitoneal injection before and after stereotactic injection of LPS (5 mg/kg). After 7 days of post-LPS injection, microglial activation and DA neuron loss were assessed by double immunostaining of Iba1 (a microglial cell marker) or CD68 (a microglial cell marker) with TH (a dopaminergic neuron marker). The results showed that LPS administration elicited an increase in the immunoreactivity of Iba1 and CD68, which was accompanied by significant TH-positive cell loss in the SN region. LPS-induced microglial activation and subsequent TH-positive cell loss were significantly attenuated by 2-DG treatment (Fig. 7b–f). It is currently well accepted that IL-1β plays a critical role in amplifying inflammatory response in the brain and contributes to neurodegeneration. Thus, we further evaluated the effect of 2-DG on IL-1β expression in SN in an LPS-induced PD mouse model. As shown in Fig. 7g, 2-DG significantly inhibited the LPS-induced increase of IL-1β expression in SN region. These results indicate that the inhibition of glycolysis ameliorates neuroinflammation and subsequent dopaminergic cell death.

### Glycolytic inhibitor 2-DG significantly ameliorates MPTP-induced behavioral deficits in mice

**Fig. 8:**
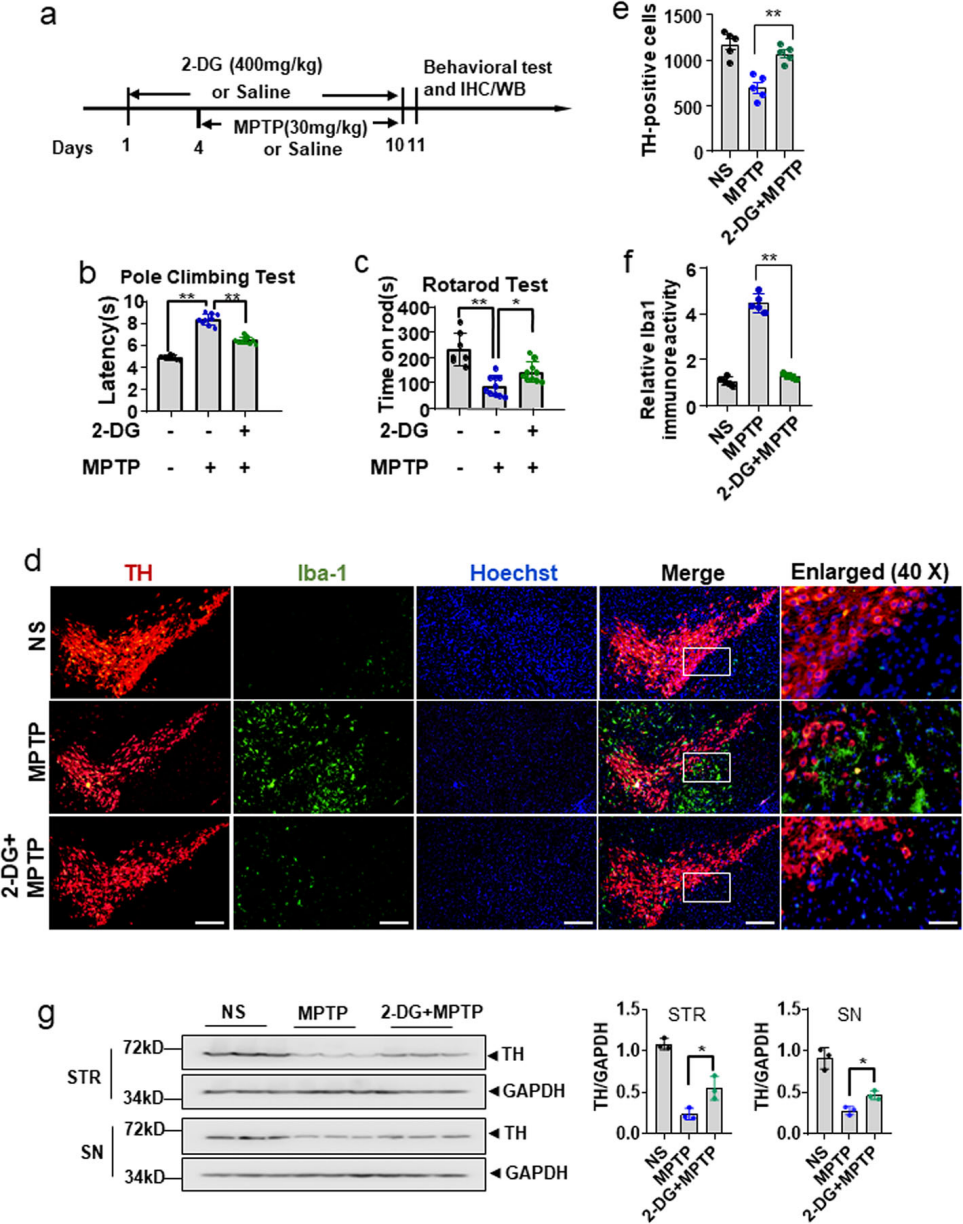
Glycolytic inhibitor 2-DG significantly ameliorates MPTP-induced behavioral deficits in mice. **a** Schematic diagram of drug administration. Mice were injected intraperitoneally with 2-DG (400 mg/kg/day) or saline (NS) for sequential 10 days. On the fourth day, mice were injected intraperitoneally with MPTP (30 mg/kg/day) or saline (NS) for 7 days. Pole climbing test (**b**) and rotarod test (**c**) were conducted on the 11th day (n=10 mice per group). **d** Double immunostaining for TH and Iba-1 in the SN. Magnification, ×10, scale bar, 100 μm (*left*). Boxed rectangular regions were enlarged (*right*). Magnification, ×40, scale bar, 25 μm. **e–f** The number of TH-positive cells was quantified manually, and the optical density of Iba1 immunoreactivity in same sections were measured using ImageJ analysis software (n=5 mice per group). The data are presented as the means ± S.E.M. *p <0.05, **p <0.01, compared to the MPTP group. **g** TH protein expression in the striatum (STR) and SN was determined by Western blotting (*left*), and the relative protein levels were quantified by densitometric analysis (*right*, n=3 mice per group). The data are presented as the means ± S.D. *p <0.05, **p <0.01, compared to the MPTP group

It is well known that damaged neurons may elicit surrounding microglial activation. In turn, activated microglia facilitate neuronal damage by releasing a variety of neurotoxic factors [2]. Therefore, inhibition of microglial cell activation may block a self-amplifying cycle of neuronal injury and microglial activation, which eventually endows neuroprotection [3]. Thus, we investigated whether 2-DG improved MPTP-induced substantia nigra neuronal cell damage and subsequent microglial activation (Fig. 8a). The results showed that pretreatment with 2-DG significantly mitigated MPTP-induced mice behavioral defects (Fig. 8b, c). In addition, immunofluorescence and Western blot analysis showed that 2-DG treatment markedly reduced TH-positive cell loss and microglial cell activation (Fig. 8d–g). We demonstrated that 2-DG treatment did not protect MPP^+^-induced MES23.5 cell death in vitro (Fig. 6b; Fig. s7b). Taken together, these results indicate that inhibition of glycolysis elicits neuroprotection by blocking the vicious cycle of dopaminergic cell damage and neuroinflammation in vivo.

## Discussion

In the present study, it was found that inhibition of aerobic glycolysis suppressed LPS-induced microglial proinflammatory gene expression at both mRNA and protein levels via the inhibition of NF-κB transcriptional activity. The mechanistic studies indicated regulation of AMPK/mTOR/IKKβ and NAD^+^/SIRT1/p65 signaling pathways may be involved in the inhibitory activity of glycolytic inhibitors on LPS-induced NF-κB transcriptional activation. Furthermore, 2-DG significantly attenuated microglial activation and dopaminergic cell loss in both LPS- and MPTP-induced PD models.

The metabolic shift from oxidative phosphorylation to aerobic glycolysis has emerged as a hallmark of the proinflammatory activation of microglial cells. However, it is largely unknown whether this reprogramming is required for the expression of proinflammatory genes in LPS-activated microglial cells. We found that the knockdown of glycolytic enzymes and application of small-molecule inhibitors both blocked LPS-induced microglial activation, suggesting that glycolysis is essential for microglial inflammatory activation. Previously, it was reported that aerobic glycolysis controlled IFN-gamma production upon the binding of GAPDH to the AU-rich elements of the 3′-untranslated regions of IFN-gamma mRNA in T cells [33]. It was also reported that GAPDH-ARE binding is critical for posttranslational control of TNF-α production in LPS-primed monocytes [34]. However, in dendritic cells, 2-DG suppressed the production of TNF-α, IL-6, and IL-12 at posttranscriptional levels through GAPDH-ARE binding independent mechanisms [17]. In our results, we found that glycolytic inhibition reduced the expression of proinflammatory genes at both mRNA and protein levels, suggesting that transcriptional mechanisms are also involved in the regulation of microglial activation by glycolysis.

During microglial activation, the expression of proinflammatory genes is mainly controlled by NF-κB and AP-1, which are key transcriptional factors that regulate the expression of a number of genes critical for inflammatory responses. After binding with LPS, TLR4 elicits TAK1 activation by recruiting adaptor molecules and protein kinases such as MyD88, IRAKs, and TRAF6, resulting in phosphorylation of IKKβ and MAPK s[35]. The activation of MAPKs, including JNK, p38, and ERK1/2, elicits transcriptional factor AP-1 activation, ultimately leading to the expression of proinflammatory genes. In the present study, we found that glycolytic inhibitors did not reduce LPS-induced MAPK phosphorylation but suppressed IKKβ phosphorylation, suggesting that TAK1 is not involved in the anti-inflammatory activity of glycolytic inhibitors in microglial cells. In addition, we also found that glycolytic inhibitors blocked both IKKβ and p65 overexpression—but not TAK1 overexpression-induced NF-κB luciferase activity. Furthermore, inhibition of glycolysis suppressed LPS-induced the phosphorylation and nuclear translocation of p65, and the phosphorylation and degradation of IκBα in microglial cells. Taken together, these results strongly suggest that IKKβ and p65 may be targets of glycolytic inhibition.

Given that TAK1 was not involved in the anti-inflammatory effects of the glycolytic inhibitors in microglial cells, another signaling molecule may be upstream of IKKβ and p65. There are several lines of evidence showing that mTOR, a downstream molecule of AMPK, is involved in the activation of NF-κB signaling pathways [28, 36, 37]. mTOR promotes NF-κB transcriptional activity by directly interacting with IKK, and rapamycin, an inhibitor of mTOR reduces proinflammatory gene expression by blocking NF-κB activation in microglial cells [38]. Our previous study also demonstrated that rapamycin significantly suppressed IKKβ phosphorylation in LPS-activated BV-2 microglial cells [28]. In the present study, we found that inhibition of glycolysis suppressed LPS-induced mTOR phosphorylation, which was consistent with the findings from previous report which indicated that 2-DG was shown to inhibit mTOR in some cancer cell lines [39]. Phosphorylation of mTOR is mainly regulated by the upstream molecule AMPK [38]. AMPK is a highly conserved protein kinase complex that is a key component of cellular energy metabolism in eukaryotes [38]. Activation of AMPK is inhibited by high levels of fructose-1,6-bisphosphate (FBP), an intermediate metabolite of glycolysis [40]. AMPK induces rapid inhibition of mTOR signaling by directly phosphorylating tuberous sclerosis complex subunit 2 (TSC2) and rapto r[38]. There is growing evidence showing that activation of AMPK significantly inhibits microglial inflammatory activation by blocking the NF-κB signaling pathway [41, 42]. AMPK is activated by the phosphorylation of Thr172 at a high ADP/ATP rati o[41]. In the present study, glycolytic inhibitors activated AMPK by increasing the ADP/ATP ratio. Activation of AMPK resulted in the inhibition of mTOR phosphorylation and IKKβ activation in microglial cells, suggesting that the AMPK/mTOR/IKKβ signaling pathway is at least partly involved in the anti-inflammatory mechanisms of glycolytic inhibition. In addition to phosphorylation, the acetylation of p65 is also crucial in regulating NF-κB transcriptional activation under inflammatory conditions [43]. The acetylation of p65 is mainly mediated by different histone acetyltransferases, including CREB-binding protein and p300 [43]. The acetylation status of p65 is also negatively controlled by histone deacetylases, including SIRT1 [29]. SIRT1, an NAD^+^-dependent protein deacetylase, can deacetylate p65 at lysine 310, thereby inhibiting NF-κB transcriptional activation [29]. SIRT1 activator resveratrol or overexpression of SIRT1 can significantly inhibit microglial inflammatory activation by suppressing NF-κB transcriptional activity [44]. In the present study, 2-DG blocked the LPS-induced decrease in the NAD^+^/NADH ratio and p65 acetylation, resulting in the inhibition of LPS-induced NF-κB activation. These results suggest that inhibition of the NAD^+^-dependent SIRT1/NF-κB pathway might contribute to the anti-inflammatory effects of glycolytic inhibition.

Although neuronal cell death is the hallmark of neurodegenerative diseases, microglial cell-mediated neuroinflammation was considered important in the pathogenesis and progression of neurodegenerative diseases, including PD [45]. Overactivated microglial cells create an inflammatory microenvironment by releasing various proinflammatory factors, ultimately contributing to neurodegenerative progression [46]. Many studies have demonstrated that inhibition of microglial activation by anti-inflammatory compounds or modulation of the expression of genes attenuated neuronal cell injuries under neurodegenerative conditions, suggesting that suppression of microglial activation may be considered a potential therapeutic approach to neurodegenerative diseases [47–49]. In the present study, we found that the glycolytic inhibitor 2-DG significantly inhibited neuronal cell death induced by conditioned media from activated microglial cells. We also found that pretreatment with 2-DG significantly attenuated dopaminergic cell death and microglial cell activation. Consistent with our study, a previous report demonstrated that 2-DG reduced MPTP-induced TH-positive cell death by increasing the expression of the stress-responsive proteins GRP78 and HSP70 in neuronal cells [20]. However, we did not find a protective effect of 2-DG on MPP^+^-induced neuronal death in vitro. On the other hand, many studies have suggested that 2-DG may inhibit cell growth or promote cell apoptosis in various tumor cells [50], suggesting that the effect of 2-DG on cell survival is dependent on the cell type or nature of the stimuli. It is suggested that stereotactic injection of LPS is able to induce loss of DA neurons. We found that 2-DG significantly ameliorated LPS-induced DA loss in vivo. Thus, it is reasonable to conclude that 2-DG ameliorates DA neuron loss in vivo by inhibiting microglia-mediated neuroinflammation.

## Conclusions

**Fig. 9:**
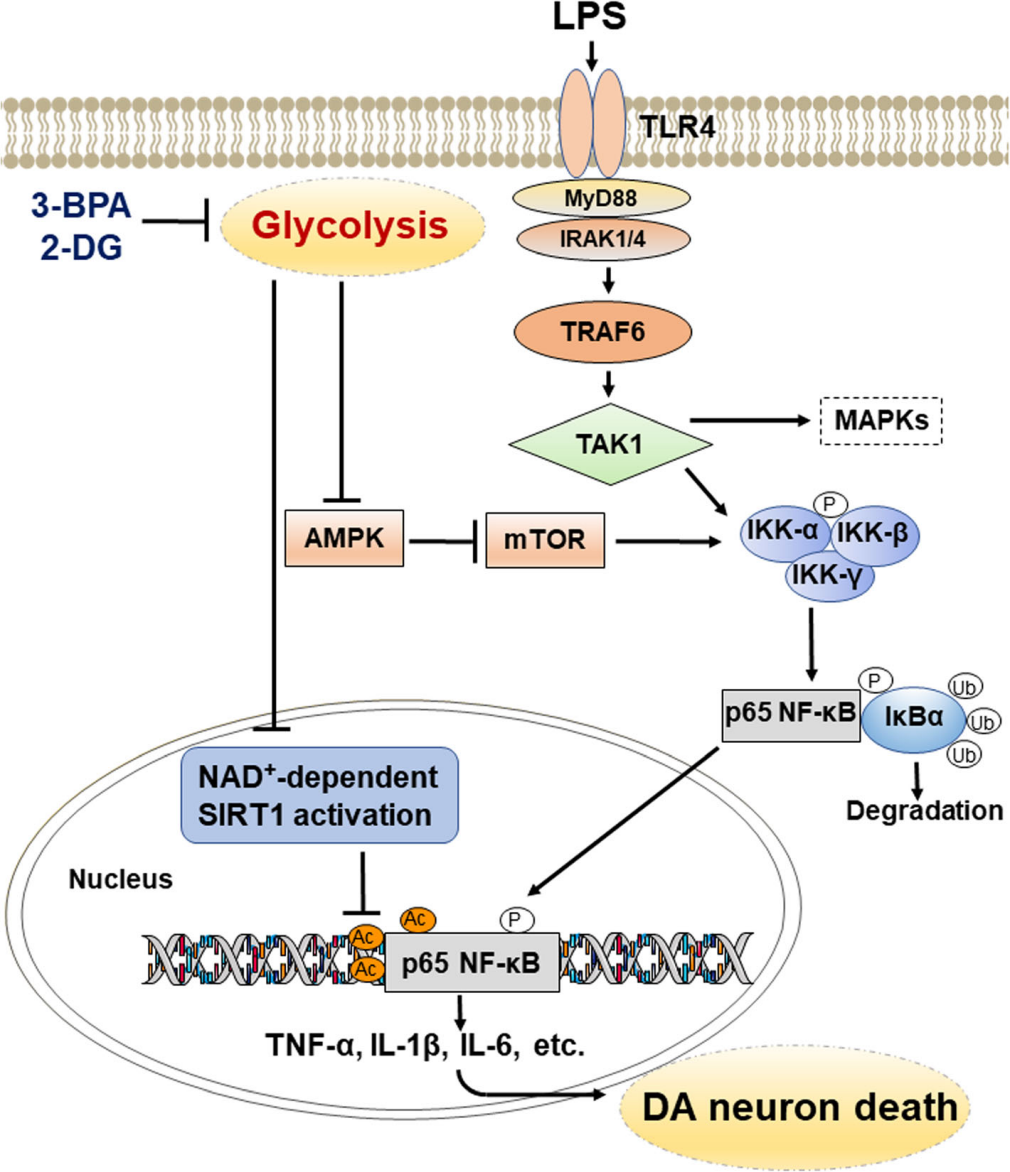
Proposed anti-neuroinflammatory mechanisms of glycolytic inhibition. Glycolytic inhibition suppressed LPS-induced activation of NF-κB signaling pathways by modulating NAD^+^/SIRT1/p65 and AMPK/mTOR/IKK/p65 signaling pathways. Therefore, inhibition of glycolysis reduced the death of DA neurons by suppressing microglial inflammatory activation

In summary, the present study demonstrated that (1) inhibition of glycolysis suppressed the expression of proinflammatory genes in LPS-activated microglial cells in vitro and in vivo; (2) inhibition of NAD^+^/SIRT1/p65 acetylation was involved in the anti-neuroinflammatory activity of the glycolytic inhibitors (Fig. 9); (3) AMPK/mTOR/IKK signaling was involved in the anti-neuroinflammatory response of the glycolytic inhibitors (Fig. 9); and (4) glycolytic inhibitor 2-DG exhibited neuroprotective effects in LPS- and MPTP- mouse PD models. Therefore, inhibition of glycolysis may be a potential therapeutic strategy for the treatment of neuroinflammation-related diseases.

## Supplementary Information

**Additional file 1.**

## Data Availability

The data that support the findings of this study are available from the corresponding author upon reasonable request.

## Abbreviations

LPS: Lipopolysaccharide
MPTP: 1-Methyl-4-phenyl-1,2,3,6-tetrahydropyridine
2-DG: 2-Dexoy-d-glucose
3-BPA: 3-Bromopyruvic acid
NF-κB: Nuclear factor-kappa B
HK II: Hexokinase 2
Glut-1: Glucose transporter 1
mTOR: Mammalian target of rapamycin
PD: Parkinson’s disease
IFN-γ: Interferon-γ
TNF-α: Tumor necrosis factor-α
NO: Nitric oxide
(PG)E_2_: Prostaglandin
IL-1β: Interleukin-1β
IL-6: Interleukin-6
ROS: Reactive oxygen species
ECAR: Extracelluar acidification rate
LDHA: Lactate dehydrogenase
iNOS: Inducible nitric oxidesynthase
COX-2: Cyclooxygenase 2
AMPK: Adenosine 5′-monophosphate (AMP)-activated protein kinase
CM: Conditional medium
IKKβ: Inhibitor of nuclear factor kappa B kinase subunit beta
SIRT1: NAD-dependent protein deacetylase sirtuin-1
PFK2: 6-Phosphofructo-2-kinase
ELISA: Enzyme-linked immunosorbent assay
TH: Tyrosine hydroxylase
MyD88: Myeloid differentiation primary response 88
RIP1: Receptor-interacting protein kinase 1
TRAF6: TNF receptor-associated factor 6
SNc: Substantia nigra pars compacta
Iba1: Ionized calcium-binding adaptor molecule 1
MAPKs: Mitogen-activated protein kinases
