# Supplementary material for: Early glycolytic reprogramming controls microglial inflammatory activation
Source: J Neuroinflammation. 2021 Jun 9;18:129. doi: 10.1186/s12974-021-02187-y (PMC8191212; doi:10.1186/s12974-021-02187-y)
Supplement: Supplementary file 1 — Additional file 1. [file 12974_2021_2187_MOESM1_ESM.docx]

**Supplementary Materials**


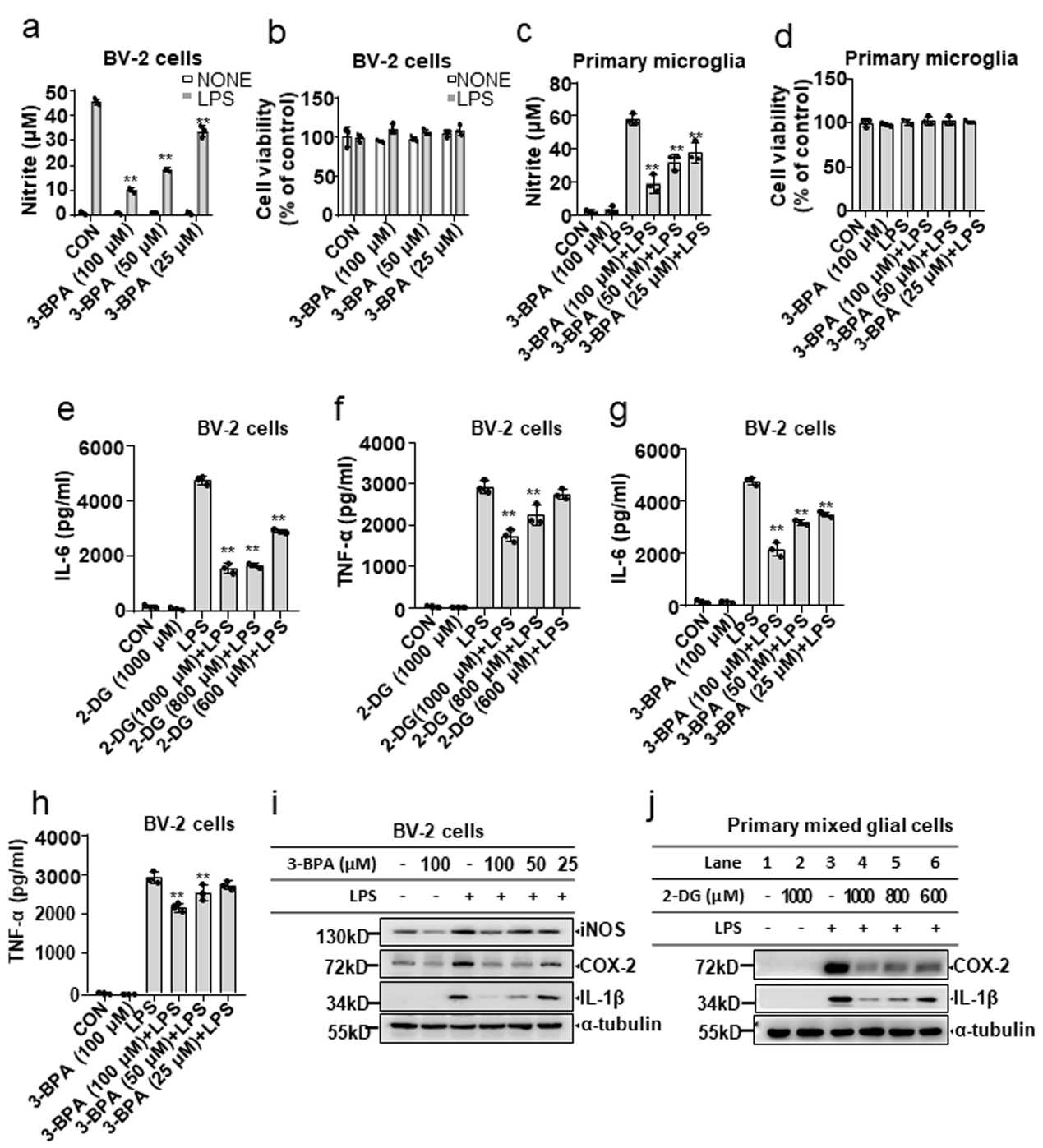


**Fig. s1** Aerobic glycolysis was required for microglial inflammatory activation. **a-d** Cells were pretreated with 3-BPA (25-100 μM) for 30 min, followed by LPS (200 ng/mL) treatment for 24 hours. The nitrite in the cell culture medium was quantified using Griess reagent (**a** BV-2 microglial cells; **c** Primary microglia). Cell viability was determined by MTT assay (**b** BV-2 microglial cells; **d** Primary microglia). **e-h** BV-2 microglial cells were pretreated with 3-BPA (25-100 μM) or 2-DG (600-1000 μM) for 30 min, followed by LPS (200 ng/mL) treatment for 24 hours. The concentrations of IL-6 (**e, g**) and TNF-α (**f, h**) in the BV-2 microglial cells culture media were analyzed by ELISAs. Data are presented as mean ± S.D. (n=3), and are representative of results obtained from three independent experiments. * *p* < 0.05, ** p <0.01, compared to LPS alone group. **i** BV-2 microglial cells were pretreated with 3-BPA (25-100 μM) for 30 min, followed by LPS (200 ng/mL) treatment for 24 hours. The expression of iNOS, COX-2 and IL-1β was determined by Western blotting. **j** Primary mixed glial cells were pretreated with 2-DG (600-1000 μM) for 30 min, followed by LPS (200 ng/mL) treatment for 24 hours. The expression of COX-2 and IL-1β in primary mixed glial cells was determined by Western blotting. Data are representative of results obtained from two or three independent experiments.


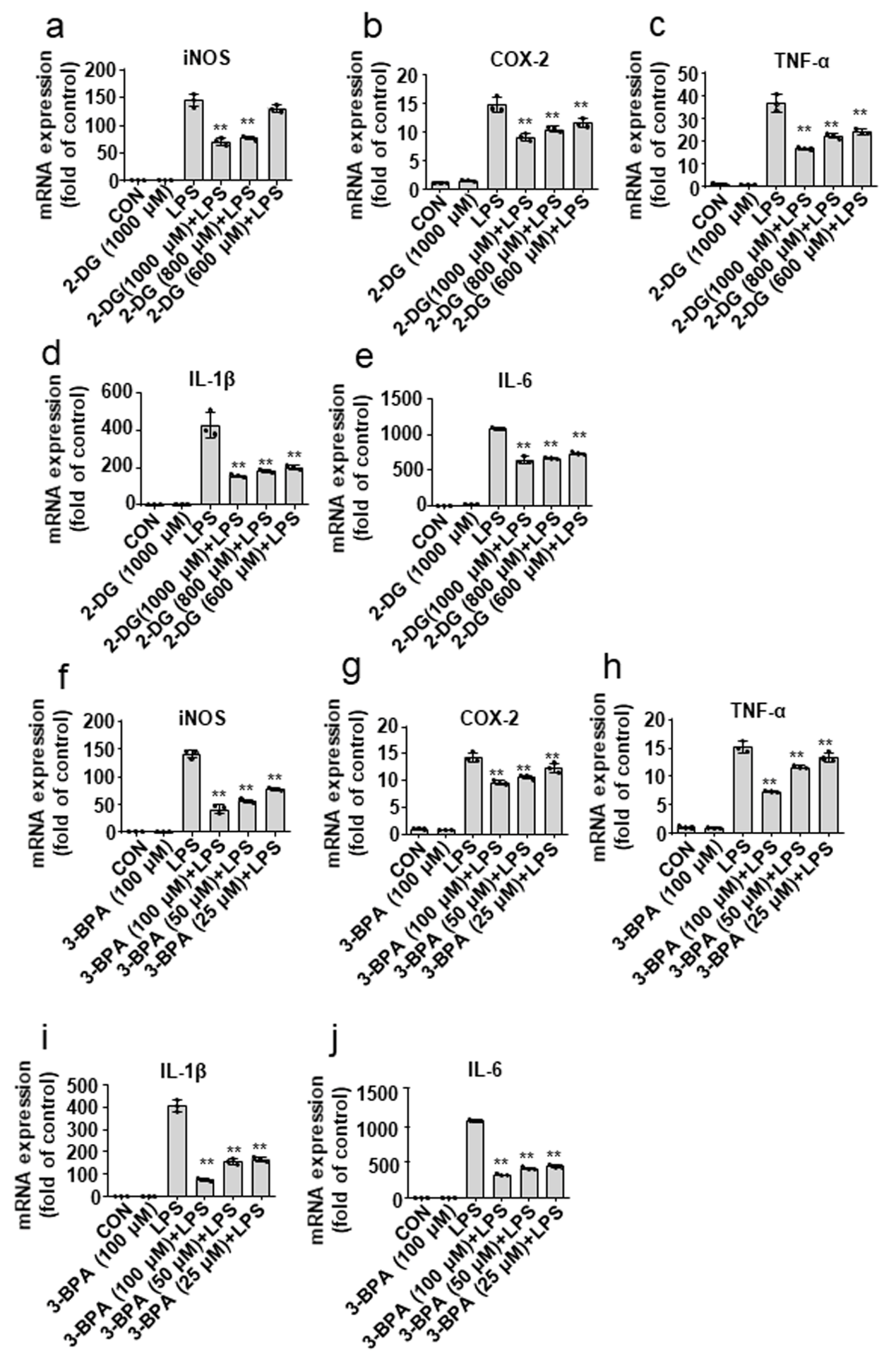


**Fig. s2** Aerobic glycolysis was required for microglial inflammatory activation. **a-j** BV-2 microglial cells were pretreated with 2-DG (600-1000 μM) or 3-BPA (25-100 μM) for 30 min, followed by LPS (200 ng/mL) treatment for 6 hours. The expression of iNOS, COX-2, TNF-α, IL-1β and IL-6 was measured by qPCR. Data are presented as mean ± S.D. (n=3), and are representative of results obtained from three independent experiments. * *p* <0.05, ** *p* <0.01, compared to the LPS group.


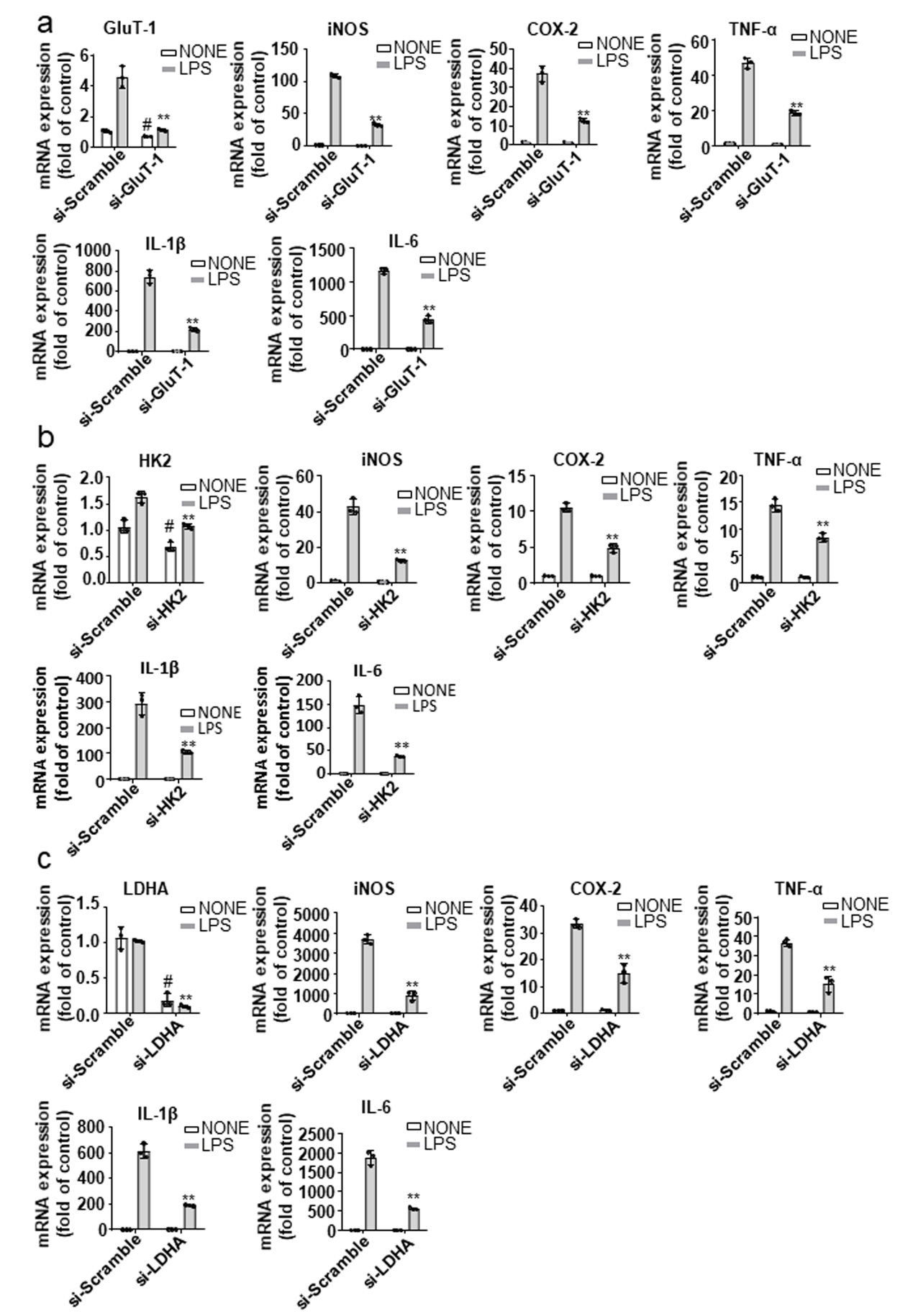


**Fig. s3** Aerobic glycolysis was required for microglial inflammatory activation. BV-2 microglial cells were transfected with si-GluT-1 (**a**) or si-HK2 (**b**), or si-LDHA (**c**) and respective scrambled siRNAs (si-Scramble). After 48 hours, BV-2 microglial cells were stimulated with LPS (200 ng/mL) for 6 hours. The knockdown efficiency was determined by qPCR analysis. Expression of iNOS, COX-2, TNF-α, IL-1β and IL-6 was measured by qPCR analysis. Data are presented as mean ± S.D. (n=3), and are representative of results obtained from three independent experiments. ** *p* <0.01, compared to the si-scramble + LPS group. # *p* <0.05, compared to si-scramble + NONE group.


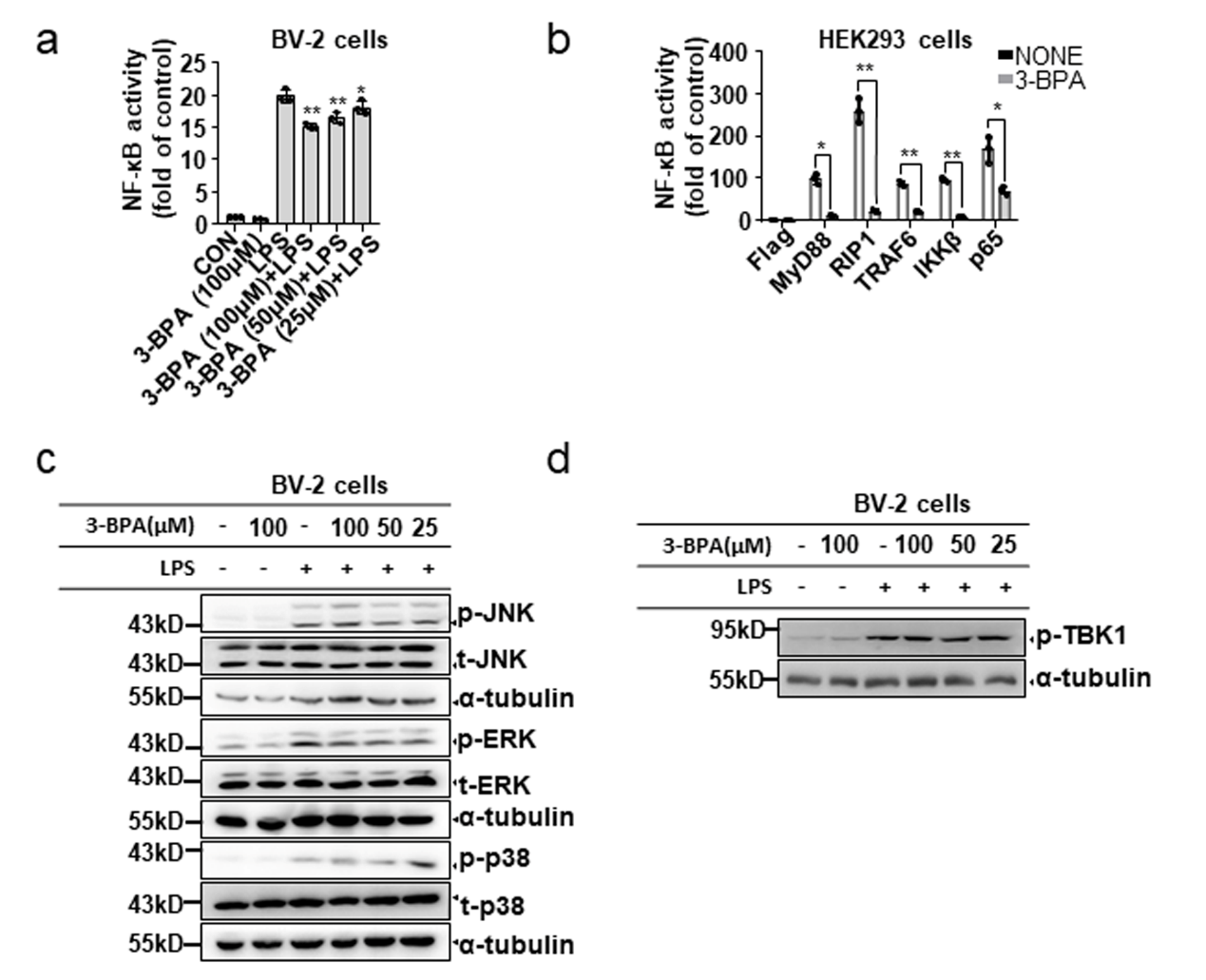


**Fig. s4** Glycolytic inhibitors suppressed the inflammatory responses by negatively regulating NF-κB signaling pathways. **a** BV-2 microglial cells stably expressing NF-κB luciferase reporter construct were pretreated with 3-BPA (25-100 μM) for 30 min, followed by LPS (200 ng/mL) treatment for 16 hours. The transcriptional activity of NF-κB was determined by luciferase reporter assay. **b** HEK 293T cells stably expressing NF-κB luciferase reporter construct were transfected with indicated plasmids. After 16 hours transfection, cells were treated with 3-BPA for 8 hours. The transcriptional activity of NF-κB was determined by luciferase reporter assay. **c** BV-2 microglial cells were pretreated with 3-BPA (25-100 μM) for 30 min, followed by LPS (200 ng/mL) treatment for 30 min. The expression of p-JNK, JNK, p-ERK, ERK, p-p38, and p38 was analyzed by Western blotting. **d** BV-2 microglial cells were treated with 3-BPA (25-100 μM) for 30 min prior to LPS stimulation for 30 min. The expression of p-TBK1 was determined by Western blotting. Data are presented as mean ± S.D. (n=3), and are representative of results obtained from three independent experiments. * *p* <0.05, ** *p* <0.01, compared to the LPS group or the NONE group.

**
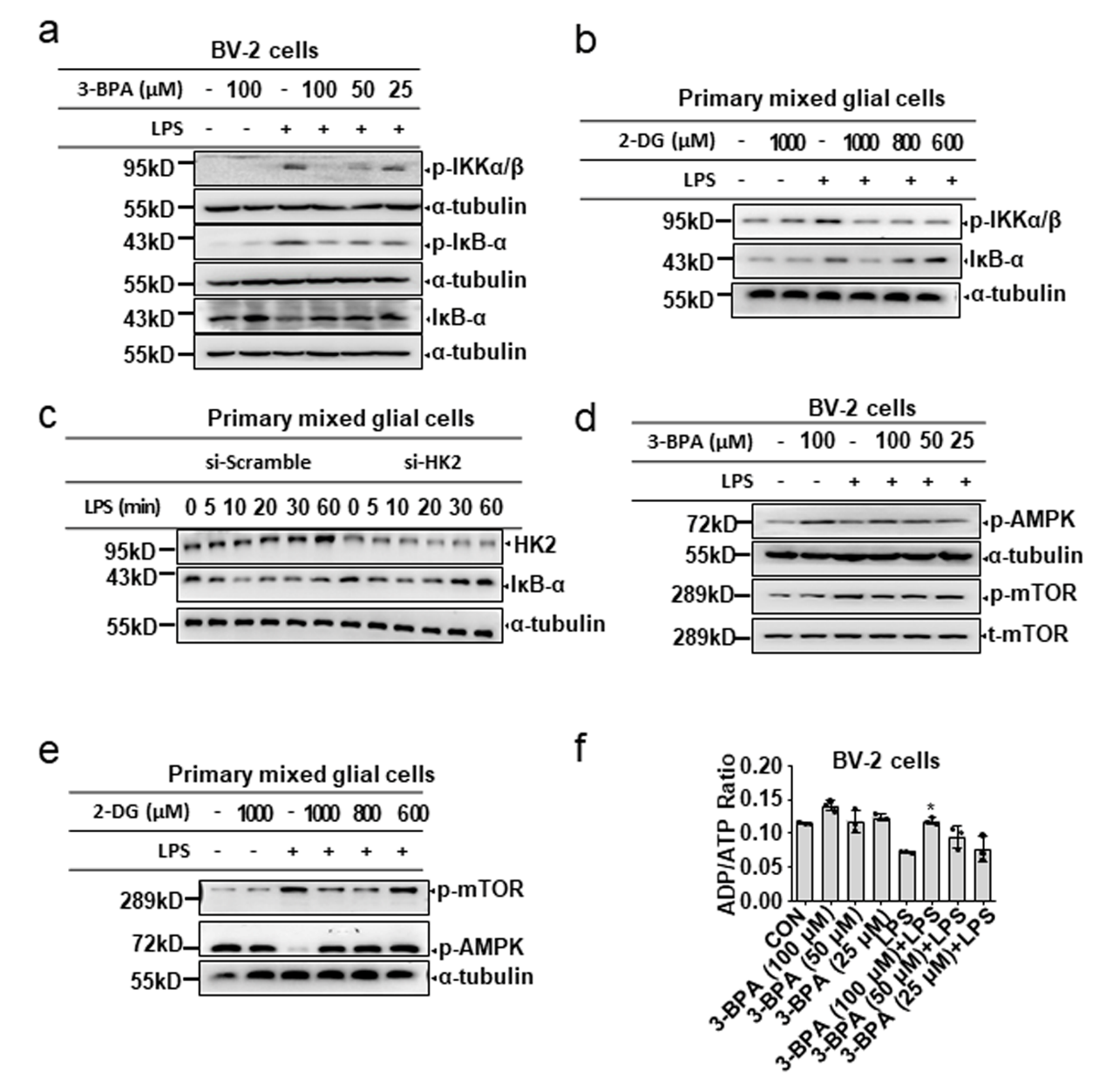
**

**Fig. s5** Glycolytic inhibitors suppressed LPS-induced IKKβ activation by modulating AMPK/mTOR signaling in microglial cells. **a** BV-2 microglial cells were pretreated with 3-BPA (25-100 μM) for 30 min, followed by LPS (200 ng/mL) treatment for 15 min. The expression of p-IKKα/β, p-IκB-α and IκB-α was measured by Western blotting (top). **b** Primary mixed glial cells were pretreated with 2-DG (600-1000 μM) for 30 min, followed by LPS (200 ng/mL) treatment for 15 min. The expression of p-IKKα/β and IκB-α was measured by Western blotting. **c** Primary mixed glial cells were transfected with scrambled siRNA or si-HK2. After 48 hours, the cells were stimulated with LPS (200 ng/mL) for indicated time points, and then the expression of HK2 and IκB-α were determined by Western blotting. **d** BV-2 microglial cells were pretreated with 3-BPA (25-100 μM) for 30 min, followed by LPS (200 ng/mL) treatment for the indicated times. After 1 hour, the expression of p-AMPK and p-mTOR was detected by Western blotting. **e** Primary mixed glial cells were pretreated with 2-DG (600-1000 μM) for 30 min, followed by LPS (200 ng/mL) treatment for the indicated times. After 1 hour, the expression of p-AMPK and p-mTOR was analyzed by Western blotting. Data are are representative of results obtained from at least two independent experiments. **f** BV-2 microglial cells were pretreated with 3-BPA (25-100 μM) for 30 min, followed by LPS (200 ng/mL) treatment. After 16 hours, the ADP/ATP ratio was calculated as described in the methods and materials section. Data are presented as mean ± S.D. (n=3), and are representative of results obtained from two or three independent experiments. * *p* <0.05, compared to the LPS group.


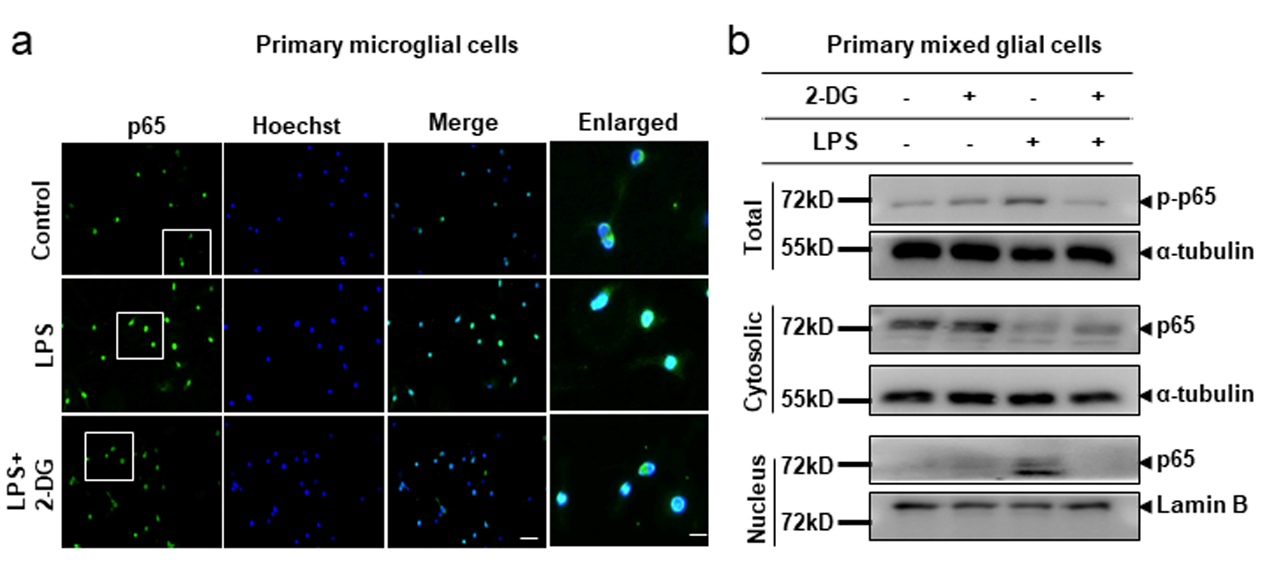


**Fig s6** 2-DG suppressed p65 nuclear translocation. **a** Primary microglial cells were plated and cultured on poly-D-lysine coated glass coverslips at a density of 2×10^4^ cells per well in 24-well plates. Cells were pre-treated with 2-DG for 30 min prior to stimulation with LPS (200 ng/mL). After 1 hour stimulation, distribution of p65 subunit of NF-κB was determined by immunofluorescence assay. Magnification, 10×, scale bar, 40 μm (*left*). Boxed rectangular regions were enlarged (*right*). **b** Primary mixed glial cells were plated at a density of 5×10^5^ cells per well in 6-well plates. Cells were pre-treated with 2-DG for 30 min prior to stimulation with LPS (200 ng/mL). After 1 hour stimulation, the expression of p65 subunit and p-p65 subunit of NF-κB in the total, cytosolic and nuclear lysates was determined by Western blotting. Data are representative of results obtained from two independent experiments.


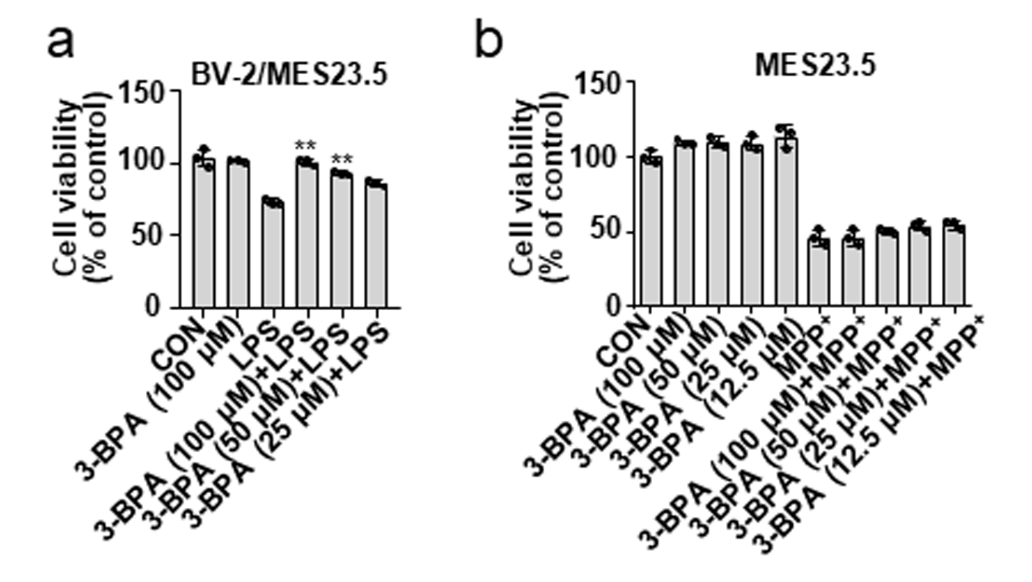


**Fig. s7** Glycolytic inhibitors reduced CM of activated microglial cell-induced MES23.5 neuroblastoma cell death. **a** BV-2 microglial cells were pretreated with 3-BPA (25-100 μM) for 30 min, followed by LPS (200 ng/mL) treatment for 6 hours. The culture medium was removed and fresh medium was added. After 24 hours of incubation, the culture medium was added to MES23.5 cells and then cultured for 24 hours. Cell viability was determined by MTT assay. **b** MES23.5 cells were treated with 3-BPA (12.5-100 μM) and MPP^+^ (1000 μM) for 24 hours. Cell viability was determined by MTT assay. Data are presented as mean ± S.D. (n=3), and are representative of results obtained from three independent experiments. ** *p* <0.01, *** *p* <0.001, compared to the LPS group.
